# The cardiac glycoside ZINC253504760 induces parthanatos-type cell death and G2/M arrest via downregulation of MEK1/2 phosphorylation in leukemia cells

**DOI:** 10.1007/s10565-023-09813-w

**Published:** 2023-06-16

**Authors:** Min Zhou, Joelle C. Boulos, Sabine M. Klauck, Thomas Efferth

**Affiliations:** 1 Department of Pharmaceutical Biology, Institute of Pharmaceutical and Biomedical Sciences, Johannes Gutenberg University-Mainz, Staudinger Weg 5, 55128 Mainz, Germany; 2 Division of Cancer Genome Research, German Cancer Research Center (DKFZ), German Cancer Consortium (DKTK), National Center for Tumor Disease (NCT), 69120 Heidelberg, Germany

**Keywords:** Cardiac glycosides, Leukemia, MEK inhibitors, Parthanatos, Synthetic derivative, Transcriptomics

## Abstract

Overcoming multidrug resistance (MDR) represents a major obstacle in cancer chemotherapy. Cardiac glycosides (CGs) are efficient in the treatment of heart failure and recently emerged in a new role in the treatment of cancer. ZINC253504760, a synthetic cardenolide that is structurally similar to well-known GCs, digitoxin and digoxin, has not been investigated yet. This study aims to investigate the cytotoxicity of ZINC253504760 on MDR cell lines and its molecular mode of action for cancer treatment. Four drug-resistant cell lines (P-glycoprotein-, ABCB5-, and EGFR-overexpressing cells, and TP53-knockout cells) did not show cross-resistance to ZINC253504760 except BCRP-overexpressing cells. Transcriptomic profiling indicated that cell death and survival as well as cell cycle (G2/M damage) were the top cellular functions affected by ZINC253504760 in CCRF-CEM cells, while CDK1 was linked with the downregulation of MEK and ERK. With flow cytometry, ZINC253504760 induced G2/M phase arrest. Interestingly, ZINC253504760 induced a novel state-of-the-art mode of cell death (parthanatos) through PARP and PAR overexpression as shown by western blotting, apoptosis-inducing factor (AIF) translocation by immunofluorescence, DNA damage by comet assay, and mitochondrial membrane potential collapse by flow cytometry. These results were ROS-independent. Furthermore, ZINC253504760 is an ATP-competitive MEK inhibitor evidenced by its interaction with the MEK phosphorylation site as shown by molecular docking *in silico* and binding to recombinant MEK by microscale thermophoresis *in vitro*. To the best of our knowledge, this is the first time to describe a cardenolide that induces parthanatos in leukemia cells, which may help to improve efforts to overcome drug resistance in cancer.

**Graphical Abstract:**

A cardiac glycoside compound ZINC253504760 displayed cytotoxicity against different multidrug-resistant cell lines. ZINC253504760 exhibited cytotoxicity in CCRF-CEM leukemia cells by predominantly inducing a new mode of cell death (parthanatos). ZINC253504760 downregulated MEK1/2 phosphorylation and further affected ERK activation, which induced G2/M phase arrest.

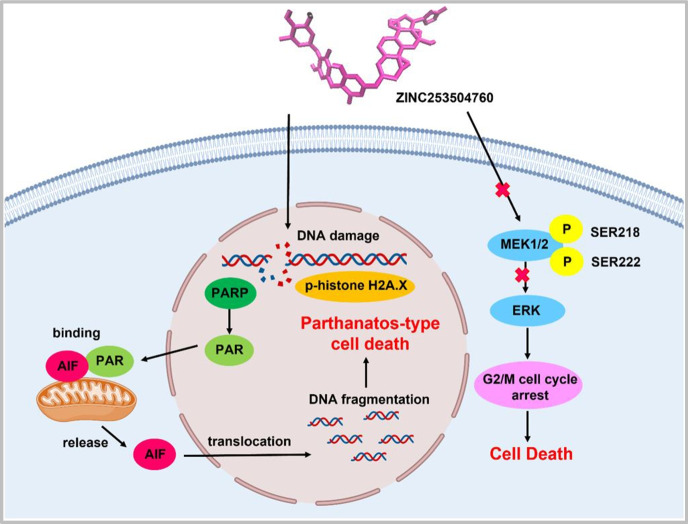

**Supplementary Information:**

The online version contains supplementary material available at 10.1007/s10565-023-09813-w.

## Introduction

Cancer is one of the leading causes of death worldwide. Although chemotherapy made great progress as a major therapeutic strategy in oncology, the development of multidrug resistance (MDR) still limits drug efficiency and the successful treatment of patients.

**Fig. 1:**
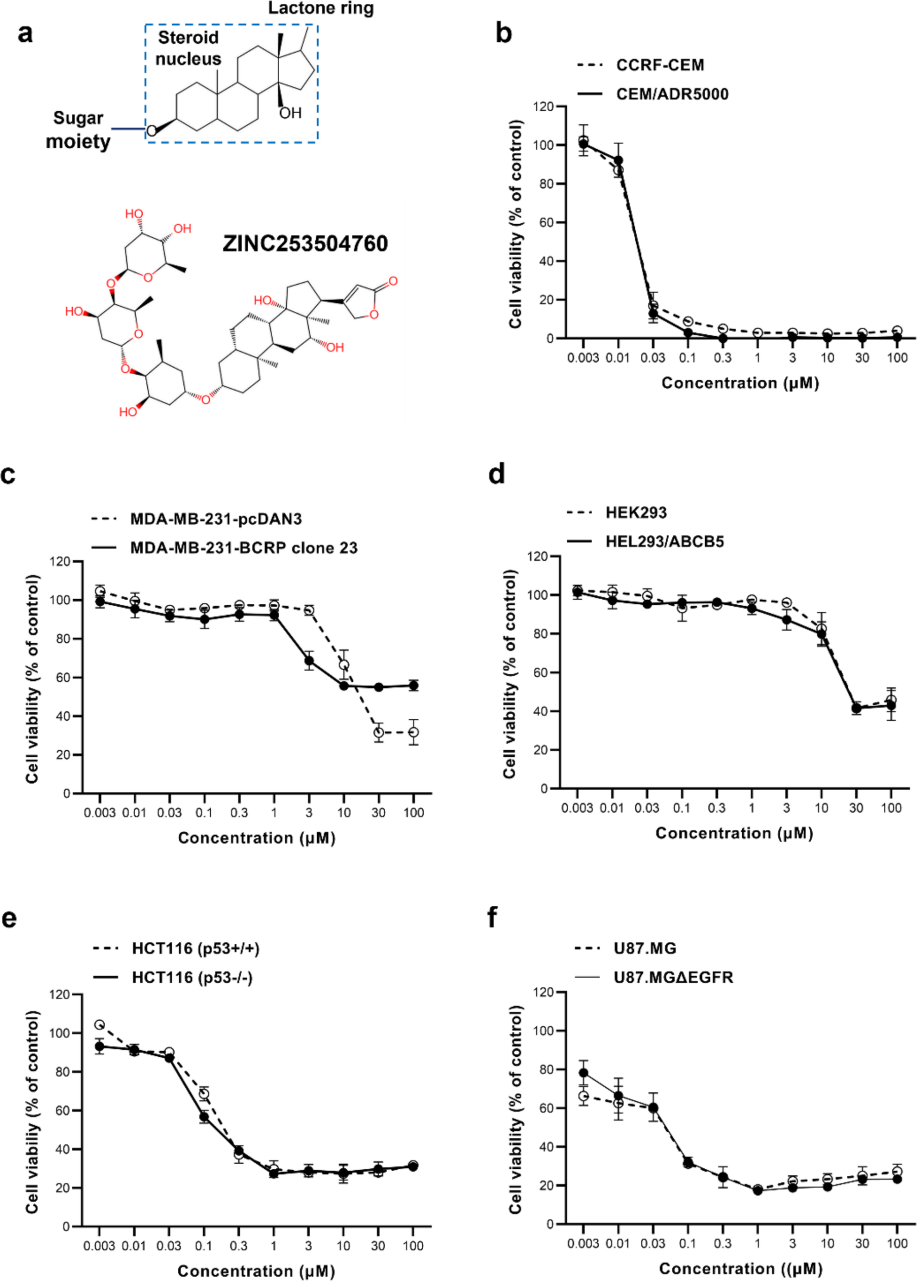
Dose-response curves of ZINC253504760 towards drug-sensitive and drug-resistant cell lines as determined by the resazurin assay. **a** Steroid nucleus, the core structure of cardiac glycoside, and the chemical structure of ZINC253504760. **b** Growth inhibition of ZINC253504760 towards CCRF-CEM and ABCB1/P-glycoprotein expressing CEM/ADR5000 leukemia cell lines. **c** Growth inhibition of MDA-MB-231-pcDNA3 and their transduced MDA-MB-231-BCRP clone 23 breast cancer cell lines. **d** Growth inhibition of HEK293 and their transduced HEK293/ABCB5 human embryonic kidney cancer cell lines. **e** Growth inhibition of HCT116 (p53^+/+^) and p53^-/-^ knockout HCT116 (p53^-/-^) colon cancer cell lines. **f** Growth inhibition of U87MG wild-type and their transfected U87MG.ΔEGFR glioblastoma cell lines. The results are mean values ± SD of three independent experiments

Cardiac glycosides (CGs) are a class of steroid-like naturally derived products (Prassas and Diamandis 2008). CG-containing herbs have been used in folk medicine by native people of the ancient Egyptian culture, and Arab physicians treated heart and malignant diseases (Gurel et al. 2017). In the year 1785, extracts from *Digitalis purpurea* containing CGs were first described for medical use in the Western world by William Withering (Bessen 1986). Nowadays, more than 100 CGs have been identified in plants and animals (Mijatovic et al. 2007). *Digitalis-*based drugs such as digitoxin and digoxin are still in clinical use as oral medications for treating heart failure and atrial arrhythmias. In the case of myocardial fibrosis, CGs bind to and inhibit Na^+^/K^+^-ATPase, allowing calcium to elevate contraction and improve myocardial contraction and cardiac pump activity (Newman et al. 2008; Prassas and Diamandis 2008). Chemically, CGs consist of a steroid core, with a sugar portion at position 3 and an unsaturated lactone ring at position 17 (Fig. 1a). The two CGs classes are cardenolides and bufadienolides, both of which have unsaturated five-or six-membered rings, respectively (El-Seedi et al. 2019a).

Since the 1980s, Stenkvist *et al*. noted that breast cancer cells from women who received *Digitalis* therapy showed a lower risk of recurrence compared with untreated patients, suggesting CGs may have strong anticancer effects (Stenkvist et al. 1979, 1982). During the past three decades, the interest in developing CG as an anticancer drug has been steadily growing. The initial anticancer mechanism of CGs is binding to Na^+^/K^+^-ATPase and altering signal transduction pathways to affect the growth of human malignant tumor cells, in particular glioblastoma, melanoma, and non-small cell lung cancer where Na^+^/K^+^-ATPase is vastly expressed (Silva et al. 2021). This makes CGs promising chemotherapeutic candidates for anticancer treatment (Ayogu and Odoh 2020). Subsequently, other modes of action of CGs have been elucidated, including activation of ERK1/2, increased expression of cell cycle inhibitor p21 (Yuan et al. 2019), inhibition of Akt and PI3K pathway, and inhibition of transcription factors such as NF-κB (Prassas and Diamandis 2008). Different types of cancer cells, such as leukemia (Zeino et al. 2015a, b; Saeed et al. 2016), breast (Li et al. 2021), melanoma (Smolarczyk et al. 2018), lung (Hu et al. 2021), pancreatic (Ha et al. 2021), liver (Reddy et al. 2019), and kidney carcinoma (Nolte et al. 2017) induced cell death upon treatment with CGs. A series of synthetic CGs analogs raised interest because some of them showed improved anticancer properties through structural modification (Ren et al. 2020; Ainembabazi et al. 2022; Wang et al. 2017). Their synthetic route, structure-activity relationship (SAR), and the screening of anticancer activity are still in progress, and their potential modes of action are not fully elucidated yet and need further investigation.

The mitogen-activated protein kinase (MAPK) cascades are key signaling pathways in transmitting signals from cell surface receptors into the inside of the cell to regulate numerous cellular activities, including cell proliferation, survival, and differentiation (Roberts and Der 2007). The Ras-Raf-MEK-ERK signaling is the most extensively studied pathway, which is aberrantly activated in nearly one-third of all human cancers (Kun et al. 2021). MEK is playing a central role in transmitting signals from Raf to ERK, making it an attractive drug target. MEK has two isoforms, MEK1 and MEK2 (Barbosa et al. 2021). The activation segments of protein kinase commonly contain phosphorylation sites. The activation of MEK1 by Raf requires the phosphorylation of two serine residues, S218 and S222 (Zheng and Guan 1994). The corresponding residues on MEK2 are S222 and S226. While both serine residues are necessary for activation, the dephosphorylation of either one would fully inactivate MEK (Roskoski 2012). MEK mutations are rare in cancer. (Ullah et al. 2022). However, constitutive MEK activation has been observed in 50 tumor cell lines (Hoshino et al. 1999), resulting in cell transformation and, eventually, tumorigenesis (Cowley et al. 1994; Mansour et al. 1994). Currently, there are four FDA-approved MEK inhibitors (MEKi), *i.e*., trametinib, cobimetinib, binimetinib, and selumetinib (Frémin and Meloche 2010; Singh et al. 2021). Tumor cells driven by mutated Raf are sensitive to MEKi *in vitro* and *in vivo*. Significantly, the combinations of Raf and MEK inhibitors (*e.g*., dabrafenib and trametinib, vemurafenib and cobimetinib) were particularly successful in BRAF^V600E^ mutant melanoma and showed better efficiency than using a Raf inhibitor alone (Flaherty et al. 2012). More importantly, MEK is the only known activator of ERK up to date, which can inhibit ERK activation and downstream molecules, thereby inhibiting cell proliferation and survival (Caunt et al. 2015). This makes MEK a prime target suppressing cell signaling. Therefore, targeting MEK is a rational solution to efficiently silence the MAPK signaling pathway in cancers.

Parthanatos is a programmed cell death mode that depends on poly(ADP-ribose) polymerase 1 (PARP1) hyperactivation independent of caspase activity. Mechanically, PARP is rapidly activated by DNA damage, such as ultraviolet light, reactive oxygen species (ROS), or alkylating agents (*e.g*., *N*-methyl-*N*’nitro-*N*-nitrosoguanidine (MNNG)). PARP produces excess poly(ADP-ribose) (PAR), which migrates from the nucleus to the cytoplasm. PAR binds to mitochondrial membrane proteins, causing apoptosis-inducing factor (AIF) to translocate from mitochondria to the nucleus, which eventually leads to large-scale fragmentation and chromatin condensation. Simultaneously, PARP overactivation causes nicotinamide adenine dinucleotide (NAD^+^) and ATP deletion, which further leads to mitochondrial depolarization. All these steps trigger parthanatos, which is distinct from apoptosis and necrosis. Parthanatos has been involved in retinal disease, ischemia-reperfusion injury, and neurodegenerative diseases including Alzheimer’s disease and Parkinson’s disease (Wang and Ge 2020). Applying PARP inhibitors to suppress parthanatos opens new treatment possibilities in these diseases. Several cancer cells such as glioma cells have a greater level of PARP and are negatively correlated with patient survival rates (Galia et al. 2012). Instead, parthanatos through hyper-activation of PARP has been induced by chemotherapies in esophageal cancer and glioma cells (Zhao et al. 2015; Ma et al. 2016), suggesting parthanatos as a promising strategy to kill cancer cells.

We recently screened a series of phytochemicals against MDR cancer cells, digitoxin and digoxin were ranked at the top with promising growth-inhibitory potential, suggesting CG compounds have the crucial benefit to anticancer effect (Khalid et al. 2022). The aim of this study was to explore the anticancer activity of a modified CG compound ZINC253504760 that could be a potential anticancer agent. We investigated the cytotoxicity of ZINC253504760 toward MDR cancer cells and studied the molecular mode of action. ZINC253504760 revealed the highest sensitivity in CCRF-CEM leukemic cells. After microarray-based mRNA profiling, we applied flow cytometry to investigate cell cycle, apoptosis and mitochondrial membrane potential. Molecular docking, western blotting and immunofluorescence were used to confirm the potential targets and the major mode of cell death.

## Material and methods

### Compound

Compound ZINC253504760 (IPUAC name: 3-[(3*S*,5*S*,8*S*,9*R*,10*S*,12*R*,13*S*,14*S*,17*S*)-3-[(2*S*,4*R*,5*R*,6*R*)-5-[(2*R*,4*R*,5*R*,6*R*)-5-[(2*S*,4*S*,5*S*,6*R*)-4,5-dihydroxy-6-methyloxan-2-yl]oxy-4-hydroxy-6-methyloxan-2-yl]oxy-4-hydroxy-6-methyloxan-2-yl]oxy-12,14-dihydroxy-10,13-dimethyl-1,2,3,4,5,6,7,8,9,11,12,15,16,17-tetradecahydrocyclopenta[a]henanthrene-17-yl]-2*H*-furan-5-one) was purchased from SPECS (Zoetermeer, Netherlands) (#SPECS AP-163/40806811). The chemical structure is shown in Fig. 1A. The stock solution (20 mM) was prepared in DMSO and stored at -20 ℃.

### Cell culture

The source and culture of cell lines used in this study were reported before (Saeed et al. 2019; Doyle et al. 1998; Rudbari et al. 2022; Dawood et al. 2020b). Two leukemia cell lines, drug-sensitive CCRF-CEM and multidrug-resistant CEM/ADR5000 were cultured in RPMI 1640 medium. Doxorubicin (5,000 ng/mL) has been added to CEM/ADR5000 cells every 14 days to maintain the resistance.

Human HEK293 embryonic kidney cells and HEK293/ABCB5, breast cancer cells MDA-MB-231-pcDNA3 and MDA-MB-231-BCRP clone 23, wild-type colon cancer cells HCT116 (p53+/+) and HCT116 (p53-/-), glioblastoma multiform cells U87MG and U87MG.∆EGFR was maintained in DMEM medium (Invitrogen, Darmstadt, Germany). Both media were supplied with 10% fetal bovine serum (FBS) and 1% penicillin-streptomycin (Invitrogen, Darmstadt, Germany). Cells were cultured at 37 °C in a humidified environment with 5% CO_2_. In addition, 400 μg/mL geneticin was continuously added to resistant cells HCT116 (p53-/-) and U87MG.∆EGFR, and 800 ng/mL geneticin for MDA-MB-231-BCRP clone 23 every two weeks. Cells in the logarithmic growth phase were used in the experiments.

### Growth inhibition assay

The growth inhibitory activity of ZINC253504760 was determined with the resazurin reduction assay (Saeed et al. 2018). A 100 μL medium containing suspension cells (1×10^4^ cells/well) or adherent cells (5 × 10^3^ cells/well) was seeded in a 96-well plate. Cells were treated with a series of concentrations of ZINC253504760 diluted in 100 μL medium. Following 72 h of incubation, 20 μL 0.01% resazurin (Promega, Mannheim, Germany) was added and incubated at 37 °C for 4 h. The fluorescence signal was measured with the Infinite M200 Pro-plate reader (Tecan, Crailsheim, Germany). The experiment was repeated in triplicates independently and with six replicates of each concentration. The growth inhibitory effect of treatment was presented as the percentage of cell viability, plotted as a dose-response curve. The fifty percent inhibition concentration (IC_50_) value was calculated from the dose-response curve using Microsoft Excel 2021. The figures were generated using Prism 8 GraphPad Software (GraphPad Software Inc., San Diego, CA, USA).

### RNA extraction

The procedure of RNA extraction has been previously reported (Mahmoud et al. 2018). One million CCRF-CEM cells were treated in duplicates with 1-fold IC_50_ of ZINC253504760 or DMSO (solvent control) and incubated for 24 h, followed by total RNA extraction with InviTrap^®^Spin Universal RNA Mini Kit (Invitek Molecular, Berlin, Germany). The lysis solution was prepared as 350 µL Lysis Solution TR containing 1% β-mercaptoethanol for each sample. The RNA concentrations were quantified by a Nanodrop spectrophotometer (Nanodrop Technologies, Wilmington, DE, USA).

### Microarray gene expression profiling and Ingenuity Pathway Analysis (IPA)

The duplicate extracted total RNA was further used in microarray hybridizations, which were carried out using Affymetrix GeneChips^®^ with human Clariom^TM^ S assays (Affymetrix, Santa Clara, CA, USA) at the Genomics and Proteomics Core Facility at the German Cancer Research Center (DKFZ, Heidelberg).

The differential gene expressions between the control and ZINC253504760-treated groups were analyzed by applying Chipster software as we previously reported (Hegazy et al. 2021; Kadioglu et al. 2021). Robust Multi-array Average (RMA) method was used to normalize the data. Genes were filtered using the percentage to exclude genes with a standard deviation of 0.5 from the gene mean. Then, the missing value were removed. The subsequent assessment of significantly deregulated genes used empirical Bayes t-test (*p* < 0.05) (accessed in July 2021).

The significantly deregulated genes were analyzed with the Ingenuity Pathway Analysis (IPA) software (Qiagen, Redwood City, CA, USA) (accessed in August 2021). The core analysis tool was used to generate the canonical pathway, cellular functions, and networks that were affected by ZINC253504760 treatment.

### qRT-PCR

The primers design and protocol were previously reported (Mahmoud et al. 2018). One microgram of extracted RNA was converted into cDNA with the Luna Script^TM^ RT SuperMix Kit (New England Biolabs, Frankfurt, Germany). The cDNA sample, both extracted from untreated cells (DMSO, 24 h) and treated cells (IC_50_, 24 h), were used for the verifications in qRT-PCR. Four genes, including two top-upregulated genes (*CD82* and *H2AC18*) and two top-downregulated genes (*HSP90AA1* and *HSP90AB1*), were chosen to validate the microarray results. Eight genes (*HIPK2, PPM1D, CDK1, Wee1, CKS1, CKS2, TP53*, and *CDK7*) involved in cell cycle regulation were selected to verify the mechanism. Primer sequences are presented in Supplementary Table S1. The real-time quantitative polymerase chain reaction (qRT-PCR) was carried out with the CFX384TM Touch Real-Time PCR Detection System (Bio-Rad, Munich, Germany). The expressions were normalized using *GAPDH* as an internal control gene. The 2^ΔΔCt^ method was applied to calculate the fold change of each gene between treated cells and untreated cells (Livak and Schmittgen 2001).

### Cell cycle analysis and detection of apoptosis

The cell cycle analysis was previously described using propidium iodide (PI) staining (Abdelfatah et al. 2020; Rudbari et al. 2022). CCRF-CEM cells (1 × 10^6^ cells/well) were treated with ZINC253504760 at 0.5 × IC_50_, IC_50_, 2 × IC_50_ and 4 × IC_50_, DMSO (solvent control) or doxorubicin (positive control), respectively. After corresponded incubation (24 h, 48 h or 72 h), cells were fixed with ethanol and stored at -20 ℃ for 24 h. Then, samples were centrifuged (4000 rpm, 10 min) and re-suspended in 500 μL cold PBS containing 20 µg/mL RNase (Roche Diagnostics, Mannheim, Germany), followed by staining with 50 μg/mL PI (Sigma-Aldrich, Darmstadt, Germany). The measurement was performed using a BD Accuri^TM^ C6 Flow Cytometer (Becton-Dickinson, Heidelberg, Germany). The results were analyzed using FlowJo software (Celeza, Olten, Switzerland). Cells were firstly gated based on forward and side scatter properties (FSC-A/SSC-A), the single cells were gated (FL2-A/FL2-H) in a linear manner to remove doublets or debris.

Annexin V/PI staining was applied to detect and quantify apoptotic and necrotic cells (Reutelingsperger and van Heerde 1997; Vermes et al. 1995). The protocol has been recently reported by us (Saeed et al. 2022; Rudbari et al. 2022). The same amount of CCRF-CEM cells was treated with ZINC253504760 at different concentrations, DMSO (negative control), or vincristine (positive control, 5 μM) for 24, 48 or 72 h. Cells were stained with Annexin V and PI (BioVersion/Biocat, Heidelberg, Germany) in the dark, and further analyzed using a BD Accuri^TM^ C6 Flow Cytometer. All experiments were performed three times.

### Fluorescence microscopy of the microtubule cytoskeleton

Briefly, human U2OS osteosarcoma cells were seeded in μ-Slide 8 Well (30,000 cells/well) (ibidi, Gräfelfing, Germany) and kept overnight to allow cells to adhere to the plate. Then cells were treated with ZINC253504760 (IC_50_, 2 × IC_50_, and 4 × IC_50_) or DMSO as a negative control. After 24 h, cells were washed with PBS, fixed with 4% paraformaldehyde, and stained with 1 µg/mL of 4’6-diamidino-2-phenylindole (DAPI, Sigma-Aldrich, Darmstadt, Germany) in the dark. Subsequently, the slides were immersed in Mounting Medium (ibidi, Gräfelfing, Germany). Imaging was carried out using an AF7000 widefield fluorescence microscope (Leica Microsystems, Wetzlar, Germany). Images were analyzed with Image J software (National Institute of Health, Bethesda, MD, USA). The methods have been described by us (Boulos et al. 2021).

### Western blotting

CCRF-CEM cells were treated with ZINC253504760 for the indicated times, cells were washed twice with PBS. Total proteins were extracted with M-PER Mammalian Protein Extraction Reagent (Thermo Fisher Scientific, Darmstadt, Germany). Nuclear and cytoplasmic proteins were extracted using NE-PER Nuclear and Cytoplasmic Extraction Reagents kit (Thermo Fisher Scientific). CER I, CER II, and NER reagents were added as the volume ratio at 200:11:100 μL following the manufacturer’s instructions. Both lysis buffer contained 1% Halt Protease Inhibitor Cocktail and phosphatase inhibitor (Thermo Fisher Scientific). Protein concentrations were quantified by a Nanodrop spectrophotometer.

Equal amounts of protein extracts (30 µg) were separated by 10% SDS-PAGE and blotted onto a polyvinylidene fluoride membrane (ROTIPVDF^®^). The membrane was blocked in TBST buffer containing 5% bovine serum albumin (BSA) for 1 h. Afterward, the membranes were incubated with specific primary antibodies (anti-p44/42 MAPK (Erk1/2) rabbit antibody (1:1000, Cell Signaling Technology, Leiden, The Netherlands), anti-phospho-p44/42 MAPK (Erk1/2)(Thr202/Tyr204) rabbit antibody (1:1000, Cell Signaling Technology), anti-MEK1/2 rabbit antibody (1:1000, Cell Signaling Technology), anti-phospho-MEK1/2 (Ser217/221) rabbit antibody (1:1000, Cell Signaling Technology), anti-AIF rabbit antibody (1:1000, Cell Signaling Technology), anti-Lamin B1 monoclonal antibody (1:10000, Proteintech, Planegg-Martinsried, Germany), anti-PAR mouse antibody (1:1000, Merck, Darmstadt, Germany), anti-caspase 3/p17/p19 polyclonal antibody (1:1000, Proteintech), anti-PARP rabbit antibody (1:1000, Cell Signaling Technology), anti-phospho-histone H2A.X (Ser139) antibody (1:1000, Cell Signaling Technology), anti-GAPDH rabbit antibody (1:1000, Cell Signaling Technology), anti-β-actin rabbit antibody (1:1000, Cell Signaling Technology), anti-p62, SQSTM1 polyclonal antibody (1:1000, Proteintech), or anti-Beclin 1 polyclonal antibody (1:1000, Proteintech)) overnight at 4 ℃. Finally, the membrane was incubated in anti-mouse IgG or anti-rabbit IgG, HRP-linked antibody (1:2000, Cell Signaling Technology) for 1 h at room temperature. Horseradish peroxidase (HRP) substrate (Luminate^TM^ Classico, Merck Millipore, Schwalbach, Germany) was used to detect the immunoreactive band. The protein was visualized by an Alpha Innotech FluorChem Q system (Biozym, Oldendorf, Germany). The bands were quantified using Image J software (National Institutes of Health). Relative protein expression was normalized to GAPDH or β-actin.

### Analysis of mitochondrial membrane potential (MMP)

To analyze the mitochondrial membrane potential, 5,5’6,6’-trtrachloro-1,1’3,3’-tetraethylbenyimidazolylcarbocyanine iodide (JC-1; Biomol, Hamburg, Germany) staining was used as previously reported (Özenver et al. 2018). Briefly, aliquots of 10^4^ CCRF-CEM cells were seeded in a 96-well plate and treated with 0.5-, 1-, 2- and 4-fold IC_50_ of ZINC253504760, or DMSO as a negative control, or vinblastine (1 μM) as a positive control, respectively, for 24 h. Cells were stained with 10 µL diluted JC-1 per well (1 µL JC-1 in stock: 9 µL medium) and incubated at 37 ℃ for 15 min in the dark. Subsequently, cells were washed with 200 µL Cell-based assay buffer (Biomol, Hamburg, Germany) and centrifuged at 400 × g for 5 min twice. Finally, cells were suspended with 100 µL Cell-based assay buffer and detected using a BD LSR Fortessa SORP equipment. JC-1 is the most specific fluorescence probe for measuring changes in mitochondrial membrane potential. J- aggregates or monomers are the two detectable forms with emissions of JC-1 and can be detected by flow cytometry (Smiley et al. 1991). In healthy cells with higher membrane potential, J aggregates emit red fluorescence at 520-570 nm and were collected using a 586/15 bandpass filter. While the fluorescence properties of the probe are altered according to the aggregation. In lower membrane potential, JC-1 is predominantly a monomer (dead cells) that emits green fluorescence at 488 nm and was collected using a 530/30 bandpass filter. All experiments were performed in triplicates. The FSC files were analyzed by the FlowJo software (Celeza).

### Immunofluorescence microscopy of AIF translocation

Three concentrations of ZINC253504760 (IC_50_, 2 × IC_50_, or 4 × IC_50_) or DMSO alone were treated in CCRF-CEM cells for 12 h. Cells were harvested and washed once with washing buffer (1% FBS in PBS) and kept on ice. Then, 10,000 cells of each sample were cytospinned on cover slides (Thermo Fisher Scientific, Dreieich, Germany). Subsequently, cells were fixed with 4% paraformaldehyde, then permeabilized with 1% Triton X-100 in PBS. Afterward, samples were kept in the blocking buffer containing 10% FBS and 1% BSA for 1 h. The primary antibody AIF (1:400, Cell Signaling Technology) was diluted in PBS and applied to the slides overnight in a humidified chamber at 4 ℃. After rinsing three times with washing buffer, the secondary antibody (1:700) was applied to the samples for 1 h in the dark at room temperature. Then, 1 µg/mL DAPI was added to the samples for 5 min to stain cell nuclei. At last, cells were rinsed five times with washing buffer and immersed in the Mounting Medium (ibidi). Images were taken by a Leica AF7000 widefield fluorescence microscope (Leica Microsystems) and analyzed using ImageJ software (National Institutes of Health).

### Single cell gel electrophoresis (comet assay)

The comet assay is one of the common methods for evaluating DNA damage. Under electrophoresis, damaged DNA or denatured cleaved DNA fragments migrate from the intact cells, creating a ‘‘comet tail’’ under the microscope. The Oxiselect^TM^ Comet Assay Kit (3-Well Slides) (Cell Biolabs/Biocat, Heidelberg, Germany) was applied to perform the comet assay as recently described by us (Elbadawi et al. 2023). Briefly, CCRF-CEM cells were plated in a 6-well plate (10^6^ cells per well). Cells were treated with ZINC253504760 at 0.088 µM, 0.15 µM, and DMSO (negative control), respectively, for 3 h. H_2_O_2_ (50 µM) as positive control was added to the cells for 1 h. Collected cells were centrifuged at 3,000 × g for 10 min. A ratio of 1:6 was used to mix cells (10^5^ cells/ml) suspended in cold PBS with melting agarose at 37 ℃. After samples were spread on comet slides and dried, the pre-chilled lysis buffer and pre-chilled alkaline electrophoresis solution buffer were applied to the slides in the dark. Slides were then placed horizontally in alkaline electrophoresis solution buffer in the electrophoresis chamber. Twenty Volts of voltage were delivered to the chamber for 20 min. After that, the slides were washed by pre-chilled distilled water twice, followed by cold 70% ethanol. Vista Green DNA dye was diluted at a ratio of 1:10,000 in TE buffer and added in the slides (100 µL/well). DNA damage was observed by EVOS digital inverted microscope (Life technologies GmbH, Darmstadt, Germany). Fifty comets in each treatment were randomly selected and analyzed by OpenComet in Image J software (National Institutes of Health). Tail DNA% was measured as a parameter for DNA damage (Gyori et al. 2014).

### Molecular docking

The PDB files of MEK1 and MEK2 were downloaded from the RCSB Protein Data Bank (PDB codes: 1S9J and 1S9I, respectively) (Ohren et al. 2004). ZINC253504760 and trametinib in SDF format were downloaded from the ZINC 15 database (ZINC253504760) and PubChem, respectively, and were converted to PDB files. In addition, considering GCs in human metabolism are normally hydrolyzed to their deglycosylated congeners by removing sugar moiety (Jortani and Valdes 1997), therefore, the deglycosylated form of ZINC253504760 was also performed by molecular docking. The *in silico* binding of ZINC253504760 and its deglycosylated form, and trametinib to MEK1 and MEK2 was carried out using AutoDock 4.2.6 (The Scripps Research Institute, CA, USA) (Saeed et al. 2018). AutoDockTools 1.5.6 was used for converting the proteins and ligands to PDBQT format. The grid box was set to cover the whole protein. Visual Molecular Dynamics (VMD) software was used to create the visualization of interactions.

### ROS detection

Briefly, CCRF-CEM cells (2 × 10^6^ cells/well) were seeded and treated with ZINC253504760 at different indicated concentrations or DMSO (negative control) for 12 h, and 24 h, respectively. Cells were harvested and washed with PBS, then resuspended in 1 mL PBS and incubated with 10 µM 2’7-dichlorodihydrofluorescein diacetate (H_2_DCFH-DA) (Sigma-Aldrich, Germany) at 37 ℃ for 30 min. 10 µL H_2_O_2_ at the stock concentration (positive control) (Sigma-Aldrich, Germany) was treated in cells during the incubation at 15 min. The measurement was performed on a BD Accuri^TM^ C6 Flow Cytometer (Becton-Dickinson). Histograms were analyzed using FlowJo software (Celeza). All the experiments were repeated three times independently. The protocol was described by us (Wu and Efferth 2015).

### Microscale thermophoresis

The ligand-protein interactions between ZINC253504760 and MEK1 or MEK2 were performed by microscale thermophoresis (MST) as described (Dawood et al. 2020a). The recombinant human MEK1 protein (Abcam, Berlin, Germany) and the MEK2 protein (Sino Biological, Beijing, China) were labeled using the Monolith^TM^ NT.115 Protein Labeling Kit BLUE-NHS (NanoTemper Technologies, Munich, Germany). Subsequently, the MEK1 protein (1530 nmol/L) and the MEK2 protein (990.1 nmol/L) were titrated against 16 different concentrations of ZINC253504760 in assay buffer. The fluorescence signal measurement was performed using a Monolith NT.115 instrument (NanoTemper Technologies) and the samples were loaded to the standard glass capillaries. The results were shown with 60% LED power and 10% MST power for MEK1, 40% LED power and 10% MST power for MEK2. The curves of ZINC253504760 binding to both proteins were generated using MO. affinity analysis software (NanoTemper Technologies), and the dissociation constant (*K*_d_) was calculated.

## Results

### Cytotoxicity assay

ZINC253504760 revealed cytotoxicity against a panel of drug-sensitive and -resistant cell lines with IC_50_ values in the range from 0.022 ± 0.002 µM (CCRF-CEM) to 25.95 ± 0.26 µM (HEK293) (Fig. 1 and Table 1). Except for MDA-MB-231-BCRP clone 23 that was cross-resistant, the other four drug-resistant cell lines did not show cross-resistance to ZINC253504760. The resistant ratios were 0.95 (CEM/ADR5000), 0.99 (HEK293/ABCB5), 0.81 (HCT116 p53^-/-^), and 0.98 (U87.MGΔEGFR), respectively. Evidently, ZINC253504760 induced cytotoxicity on both leukemia cells and glioblastoma cells in the nanomolar range.

**Table 1:** IC_50_ values of ZINC253504760 in different drug-sensitive and drug-resistant cell lines

Cell lines	IC_50_ (μM)	Resistance ratio
CCRF-CEM	0.022 ± 0.002	0.95
CEM/ADR5000	0.021 ± 0.001	
MDA-MB-231-pcDNA3	18.76 ± 3.50	> 5.33
MDA-MB-231-BCRP clone 23	> 100	
HEK293	25.95 ± 0.26	0.99
HEK293/ABCB5	25.57 ± 1.52	
HCT116 (p53^+/+^)	0.22 ± 0.03	0.81
HCT116 (p53^-/-^)	0.18 ± 0.03	
U87MG	0.055 ± 0.004	0.98
U87MG.ΔEGFR	0.054 ± 0.01	

Among the panel of cell lines tested, ZINC253504760 exerted the most lethal effect with the lowest IC_50_ value in CCRF-CEM cells, we used CCRF-CEM as a model to explore the molecular modes of action of ZINC253504760.

### Microarray hybridization and pathway analysis

Gene expression analysis was performed to investigate the potential mode of action of ZINC253504760. Therefore, total RNA was isolated to conduct transcriptome-wide microarray assays after CCRF-CEM cells were exposed to ZINC253504760 at the IC_50_ value (0.022 µM) or DMSO as negative control for 24 h. The genes that were differentially expressed were further subjected to IPA for signaling pathway analysis.

**Fig. 2:**
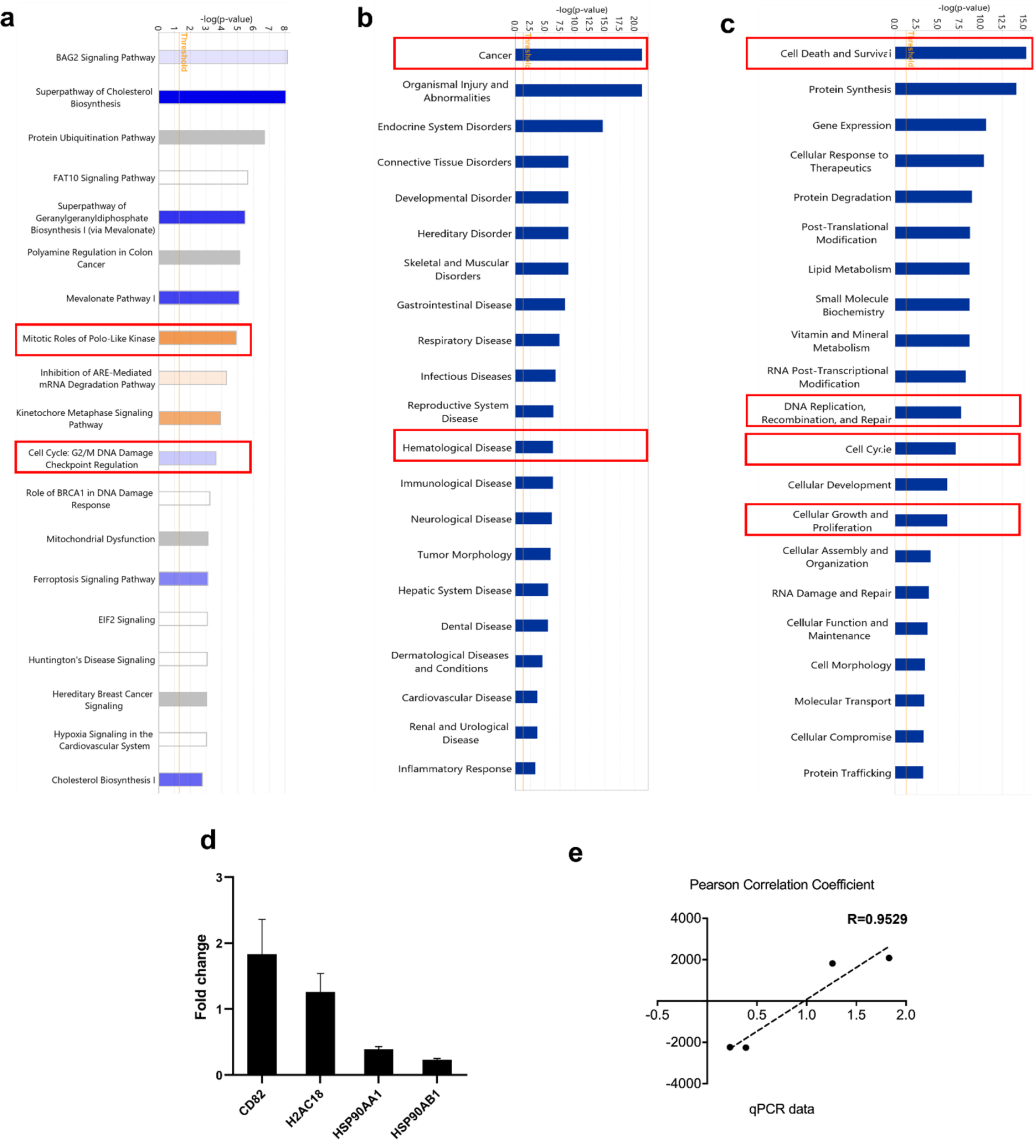
Microarray gene expression profiling and Ingenuity Pathway Analysis (IPA) in CCRF-CEM cells treated with ZINC253504760. **a** Top canonical, **b** disease, and** c** cellular functional pathways affected by ZINC253504760 were marked by the red boxes. **d** Technical verification of four selected genes by qRT-PCR analysis in CCRF-CEM cells treated with IC_50_ of ZINC253504760 for 24 h. **e** Person correlation coefficient of microarray and qRT-PCR data

IPA predicted that the “mitotic role of polo-like kinase” and “cell cycle: G2/M DNA damage” could be potential molecular mechanisms among the panel of canonical pathways (Fig. 2a). “Cancer and hematological disease” appeared as important diseases affected by ZINC253504760 (Fig. 2b). Notably, the top cellular function in Fig. 2c indicated that ZINC253504760 may affect “cell death and survival”, “DNA replication, recombination, and repair”, “cell cycle”, as well as “cellular growth and proliferation”. Moreover, there were a total of 916 genes that were deregulated between treated and untreated cells according to the expression fold change values. Among them, 368 genes were downregulated, while 548 genes were upregulated (Supplementary Tables S2 and S3). The technical verification was quantified with four selected genes (*CD82*, *H2AC18*, *HSP90AA1*, and *HSP90AB1*) with real-time RT-PCR to validate the microarray results (Fig. 2d). The top upregulated genes *CD82* and *H2AC18* in the microarray data were upregulated in qRT-PCR. *HSP90AA1* and *HSP90AB1*, the top two downregulated genes in the microarray data also showed downregulation in qRT-PCR. The correlation coefficient (R) between mRNA expression values measured by microarray and qRT-PCR was 0.95 (Pearson correlation test), which indicated a high correlation between the two methods (Fig. 2e).

**Fig. 3:**
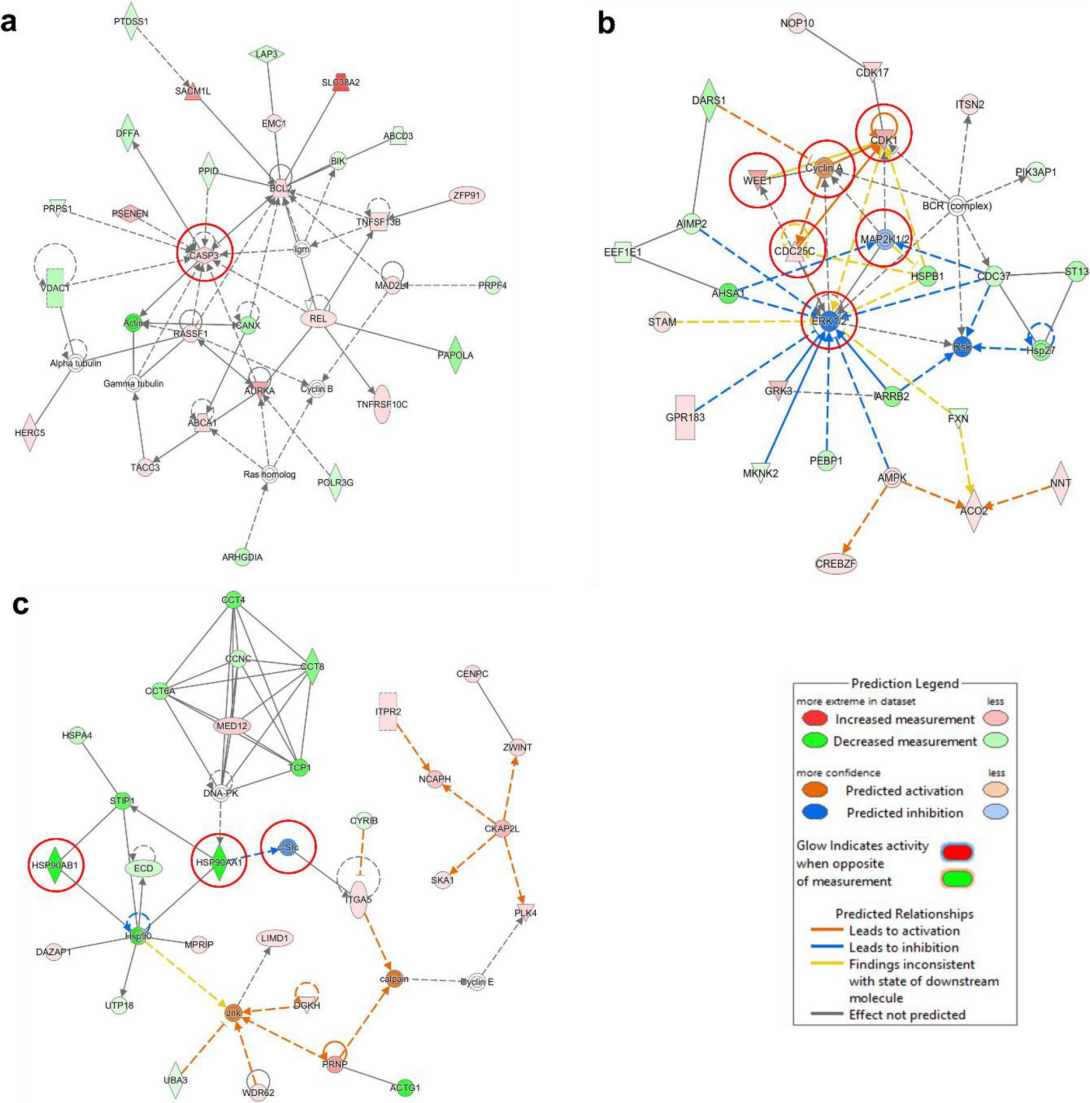
Prediction of functional networks by transcriptome-wide microarray and IPA analysis of ZINC253504760-treated CCRF-CEM cells. **a** The red circles highlight the upregulation of caspase 3, **b** The red circles highlight the genes related to the cell cycle linked with the downregulation of *MEK1/2* and *ERK*, and **c** The red circles highlight the downregulation of *Src* linked with the downregulation of *HSP90AA1* and *HSP90AB1*

The IPA-based network analysis revealed that caspase 3 expression was increased (Fig. 3a). Fig. 3b shows the increased expression of the cell cycle biomarkers including *CDK1*, *Wee1*, *Cyclin A*, and *Cdc25c*, while *MAP2K1/2* (*MEK1/2*) and *ERK1/2* were downregulated. Therefore, we conducted cell cycle and apoptosis experiments, and explored the cell death mechanisms of ZINC253504760. We chose MAP2K1/2 (MEK1/2) to further investigate whether these two proteins are targets of ZINC253504760. Moreover, Fig. 3c shows the network of top downregulated genes *HSP90AA1* and *HSP90AB1* associated with the decreased expression of *c*-*Src*.

### Cell cycle analysis

**Fig. 4:**
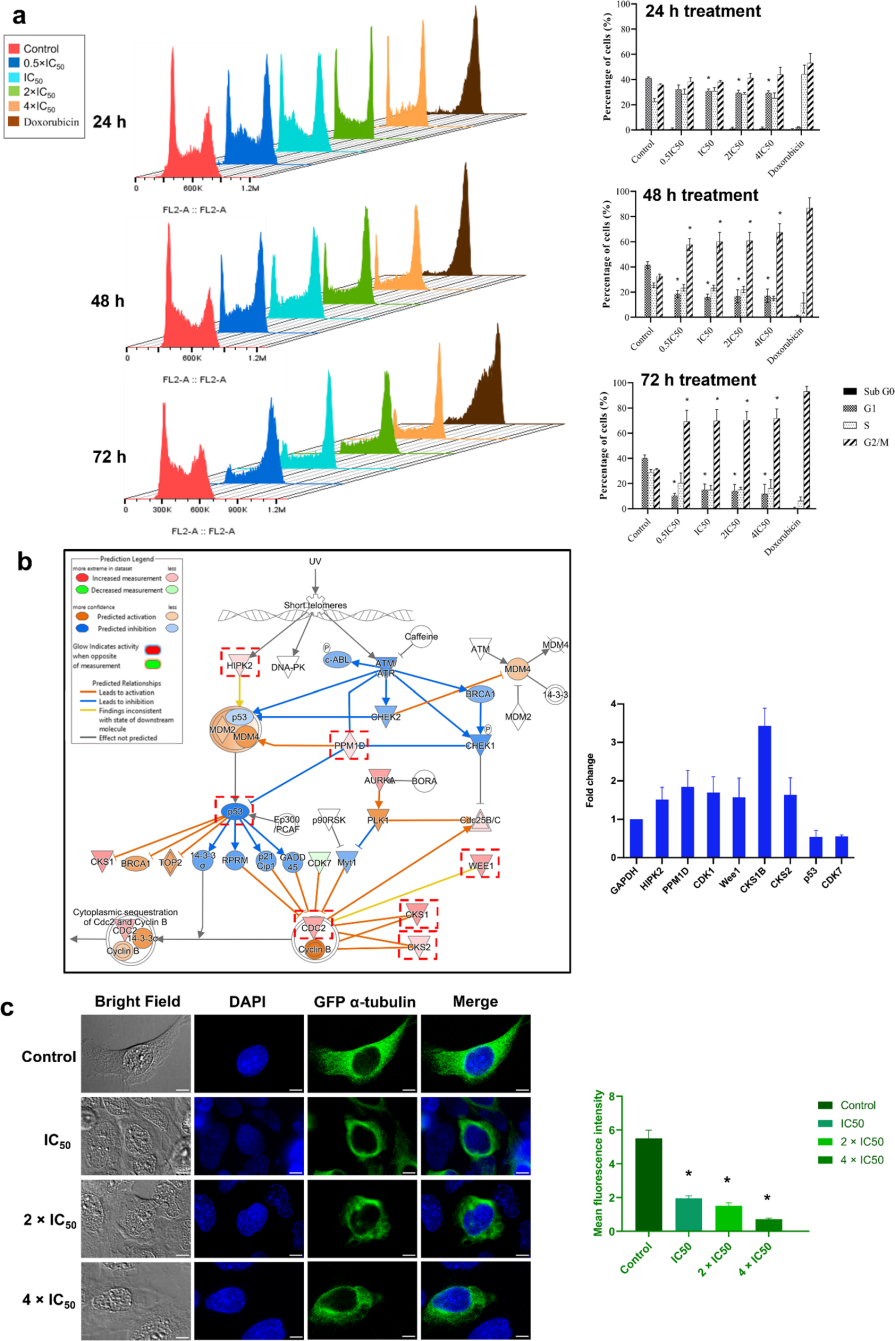
Cell cycle analysis and influence on microtubules. **a** Flow cytometric cell cycle analysis in CCRF-CEM cells treated with different concentrations of ZINC253504760, or DMSO (control), or doxorubicin (positive control) for 24, 48, or 72 h. **b** The cell cycle genes (red dotted boxes) predicted from transcriptome-wide microarray and IPA treated with ZINC253504760 after 24 h for biological verification by qRT-PCR. The results are represented as mean values ± SD of three independent experiments. **c** Immunofluorescence analysis of U2OS cells treated with ZINC253504760, or DMSO (control), and stained for DAPI (blue) and α-tubulin-GFP (green) after 24 h. The graph shows the mean fluorescence intensity of U2OS cells expressing α-tubulin-GFP tubulin. Statistics analysis was done by paired student’s t-test, * *p* < 0.05, if compared with untreated cells

To study cell cycle arrest, CCRF-CEM cells were treated with ZINC253504760 and incubated for three different timepoints. Fig. 4a shows that after 24 h treatment, the G2/M phase peak was elevated at all concentrations tested. After 48 and 72 h treatment, the arrest of cells in the G2/M phase was even more evident, and the increased percentages of the G2/M cell fraction were statistically significant compared to untreated cells (*p* < 0.05). Therefore, the flow cytometric analyses verified the IPA prediction of G2/M cell cycle arrest.

To further confirm this result, 8 genes of the G2/M arrest pathway that were deregulated in the microarray analysis were selected for biological verification using qRT-PCR. As shown in Fig. 4b, *HIPK2, PPM1D, CDK1, Wee1, CKS1,* and *CKS2* were upregulated, whereas *TP53* and *CDK7* were downregulated compared with housekeeping gene *GAPDH*. These results were consistent between qRT-PCR and microarray data.

### Influence on microtubules

The G2/M arrest caused by ZINC253504760 raises the question of how this compound impacts on the microtubules. Tubulin Alpha 1b (TUBA1B) is a microtubule protein (Liu et al. 2017). A characteristic microtubule pattern was visible in the human U2OS cell line that endogenously expresses the fluorescent fusion protein (GFP-TUBA1B, Green Fluorescent Protein (GFP) gene attached to the genomic TUBA1B gene). Therefore, U2OS cells were treated with different concentrations of ZINC253504760. Fig. 4c illustrates the effect of ZINC253504760 observed on the cellular microtubule network. In untreated U2OS cells, the microtubules extended continuously in the cytoplasm and polymerized to form an extensive intracellular network in addition to the nucleus. By contrast, ZINC253504760 significantly disrupted microtubule distribution and reduced the mass of the microtubule network. Specifically, the reduced microtubules aggregated around the nucleus, but the aggregation diminished with increasing concentrations of the treatment, finally resulting in dense microtubules being less present in the cell periphery. The thickness of microtubules weakened compared to untreated cells. It is the same effects as vincristine (postitive control) as we reported (Khalid et al. 2022). Hence, these results further strengthened the observation that ZINC253504760 induced G2/M phase arrest in the cell cycle.

### Apoptosis and autophagy detection

**Fig. 5:**
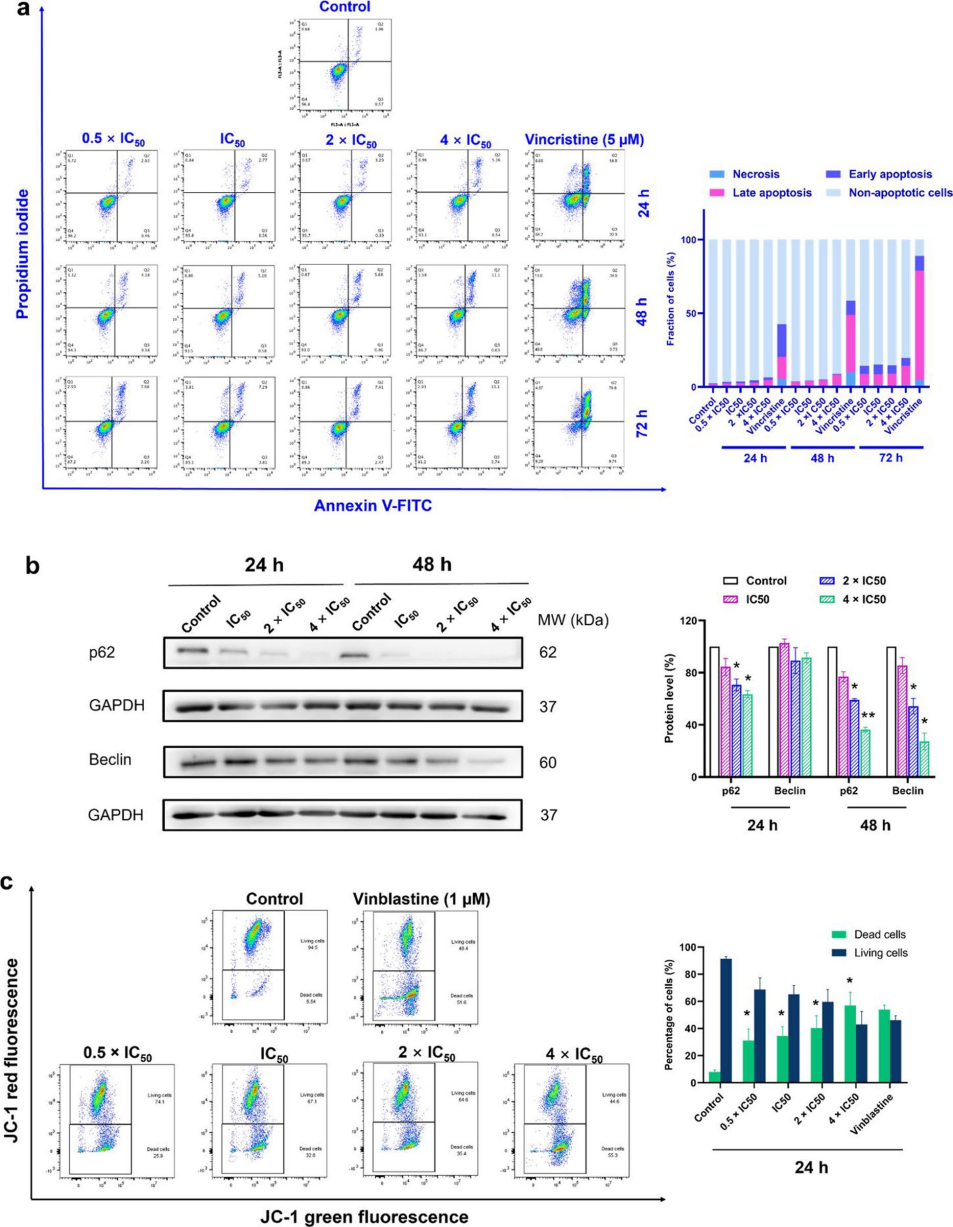
Assessment of apoptosis, autophagy and mitochondrial membrane potential. **a** Flow cytometric analysis in CCRF-CEM cells treated with different concentrations of ZINC253504760, or DMSO (control) or vincristine (positive control) for 24, 48 or 72 h. The graph shows the mean fraction of CCRF-CEM cells. The data represent as mean ± SD of three independent experiments. **b** Western blot analysis of the proteins involved in autophagy in CCRF-CEM cells treated with different concentrations of ZINC253504760 for 24 or 48 h. Data represent relative expression intensity to GAPDH. Statistics analysis was done by paired student’s t-test, * *p* ≤ 0.05, ** *p* ≤ 0.001, if compared to DMSO untreated cells. Data represent as mean values ± SEM of three independent experiments. **c** Representative images of JC-1 fluorescence with flow cytometry of mitochondrial membrane potential in CCRF-CEM cells treated with different concentrations of ZINC253504760 or DMSO (control), or vinblastine (positive control) for 24 h. Statistical results of the dead cells defined as MMP collapse after 24 h treatment. Statistics analysis was done by paired student’s t-test, * *p* ≤ 0.05, if compared to DMSO untreated cells. The statistical analysis shows mean values ± SD of three independent experiments

As cell death and survival appeared in IPA as the first cellular function, we aimed to investigate the mode of cell death induced by ZINC253504760. Apoptosis by annexin V-PI staining on the flow cytometer was first investigated. As shown in Fig. 5a, the percentage of late apoptotic cells showed a slight increase in a time- and concentration-dependent manner after treating CCRF-CEM cells with ZINC253504760 (0.5 × IC_50_, IC_50_, 2 × IC_50_, and 4 × IC_50_) for 24 h, 48 h, or 72 h, respectively. At 48 h, the late apoptotic cells were in the range of 4.18%-11.1%. At 72 h, the fractions of late apoptotic cells were 7.98%-13.1%. Vincristine induced late apoptotic cell death with 14.3% after 24 h treatment, subsequently increased to 74.2% after 72 h. It can be clearly seen that the fraction of late apoptotic cells treated with ZINC253504760 was not significant at all times and concentrations tested. More than 80% of cells were non-apoptotic.

Next, we investigated whether autophagy was related to cell death, and the expression level of p62 and Beclin was measured by western blotting. Fig. 5b shows that p62 and Beclin were both downregulated. Especially after 48 h, the decline of both biomarkers was more significant.

Therefore, these results did not provide evidence that apoptosis or autophagy were the predominant modes of cell death, and other cell death modes may contribute to the cytotoxicity observed in the resazurin assays with ZINC253504760.

### *Assessment of mitochondrial membrane potential*

Mitochondria are essential for ATP generation for life and are the key players of cell death under cell stress conditions. Even if the major form of cell death regulated by mitochondria is apoptosis, other types of cell death also have been implicated with mitochondria (Bock and Tait 2020). Therefore, we investigated the MMP to understand whether it is affected. CCRF-CEM cells were treated with different concentrations of ZINC253504760 or vinblastine (positive control, 1 µM) and measured by a flow cytometry after 24 h. As shown in Fig. 5c, the percentages of dead cells, which shift from red fluorescence (unaltered potential) to green fluorescence (defective potential), were in a range of 31.1%-57.0%. All treatments showed significant percentages of dead cells compared with untreated cells (*p* < 0.05). Especially, the 4 × IC_50_ treated sample showed a comparable effect (57.0%) to vinblastine (53.9%). Hence, we conclude that ZINC253504760 caused mitochondrial dysfunction leading to cell death.

### Western blot analysis of parthanatos

**Fig. 6:**
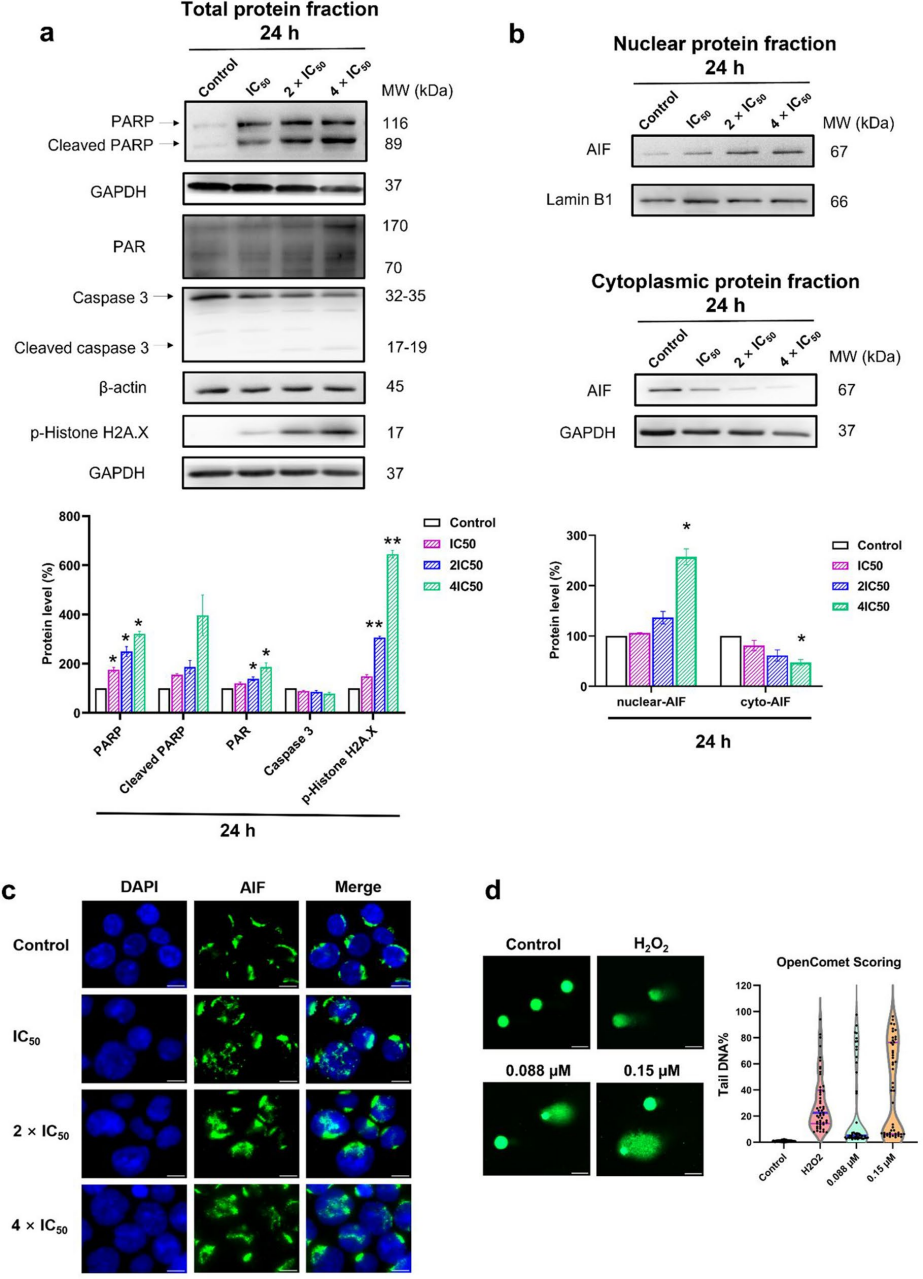
Induction of parthanatos as major mode of cell death by ZINC253504760. **a** Western blot analysis in total protein of parthanatos-related biomarkers (caspase 3, p-Histone H2A.X, PARP, and PAR) treated with different concentrations of ZINC253504760 for 24 h in CCRF-CEM cells. **b** The expression of AIF in the nucleus and the cytoplasm. Statistics analysis was done by paired student’s t-test, * *p* ≤ 0.05, ** *p* ≤ 0.001, if compared to DMSO untreated cells. The bars represent the mean values ± SEM of three independent experiments. **c** Detection of AIF translocation from the cytoplasm to the nucleus detected by immunofluorescence microscopy. CCRF-CEM cells treated with different concentrations of ZINC253504760 or DMSO (control) for 12 h and stained with antibody AIF to visualize AIF protein. Nuclear AIF (green) translocated into the nucleus (blue) was obvious. **d** Detection of DNA damage by alkaline comet assay in CCRF-CEM cells**.** Cells incubated in different concentrations with ZINC253504760 for 3 h and 50 µM of H_2_O_2_ (positive control) for 1 h. The parameter tail DNA% was measured from 50 randomly selected cells shown in the violin plot.

To investigate other forms of cell death, we first examined caspase 3 since the functional network in IPA software showed its upregulation (Fig. 3a). As depicted in Fig. 6a, caspase 3 expressions appeared but not its cleaved form, which means the mode of cell death was caspase 3-independent. Therefore, we further focused on parthanatos as a novel caspase-independent mode of programmed cell death. The expressions of biomarkers in the parthanatos pathway were examined. As shown in Fig. 6a, the expression level of PARP (116 kDa) significantly increased upon increasing ZINC253504760 concentrations. PAR also showed an increased expression. Then, we measured the nuclear and cytoplasmic AIF localization (Fig. 6b). Notably, the AIF expression decreased in the cytoplasm while increasing in the nucleus compared with the control group. Moreover, the level of p-histone H2A.X increased in a dose-dependent manner. In addition, ZINC253504760 also led to increased PARP cleavage (89 kDa), which appeared to be inconsistent with the absence of cleaved-caspase 3 expressions, we give discussions below. Taken together, these results suggest that ZINC253504760 caused parthanatos as predominant form of cell death.

### Immunofluorescence microscopy of apoptosis-inducing factor translocation

AIF is released from mitochondria and rapidly translocated to the nucleus, where it induces large-scale DNA fragmentation (50 kb) and chromatin condensation. This translocation can be captured by confocal immunofluorescence microscopy. Fig. 6c indicates that AIF was almost all extensively localized at the cytoplasm of control CCRF-CEM cells. However, if cells were treated with ZINC253504760 with IC_50_, 2 × IC_50_, or 4 × IC_50_ for 12 h, AIF accumulated in the nucleus, which was consistent with the western blotting results. These AIF translocation findings further confirmed that ZINC253504760 induced a parthanatos-type cell death.

### Single cell gel electrophoresis (comet assay)

AIF enters the nucleus, triggers cell death by binding to DNA and leads to DNA fragmentation. For this reason, we examined DNA damage at the level of individual cells by the alkaline comet assay. ZINC253504760 induced comet tails in CCRF-CEM cells upon treatment with different concentrations for 3 h (Fig. 6d). The median value (percentage of tail DNA) from 50 randomly selected cells was 5.2% with a range from 2.4% to 97.5% at a concentration of 0.088 µM. Only a few cells were damaged. Upon treatment with 0.15 µM, the median value reached 39.4% with a range from 3.9% to 96.2%, evidencing that DNA damage and the number of damaged cells increased in a concentration-dependent manner. This finding further supports the hypothesis that ZINC253504760 caused parthanatos and implies that eventually the cells die from large-scale DNA damage.

### Assessment of oxidative stress

ROS are well recognized as DNA damage mediators. Since ZINC253504760 resulted in a pathanatos, G2/M phase arrest, collapse of mitochondrial membrane potential and a minor fraction of apoptosis, we further investigated whether they were induced via ROS generation. Compared with H_2_O_2_ that showed a high fold change of ROS production, ZINC253504760-treated samples did not show ROS generation at 12 h or 24 h (Supplementary Fig. S4). Therefore, ZINC253504760 did not induce ROS-dependent mitochondrial apoptosis pathway, and the induction of pathanatos and G2/M phase arrest were not ROS-dependent.

### Western blotting of MEK

**Fig. 7:**
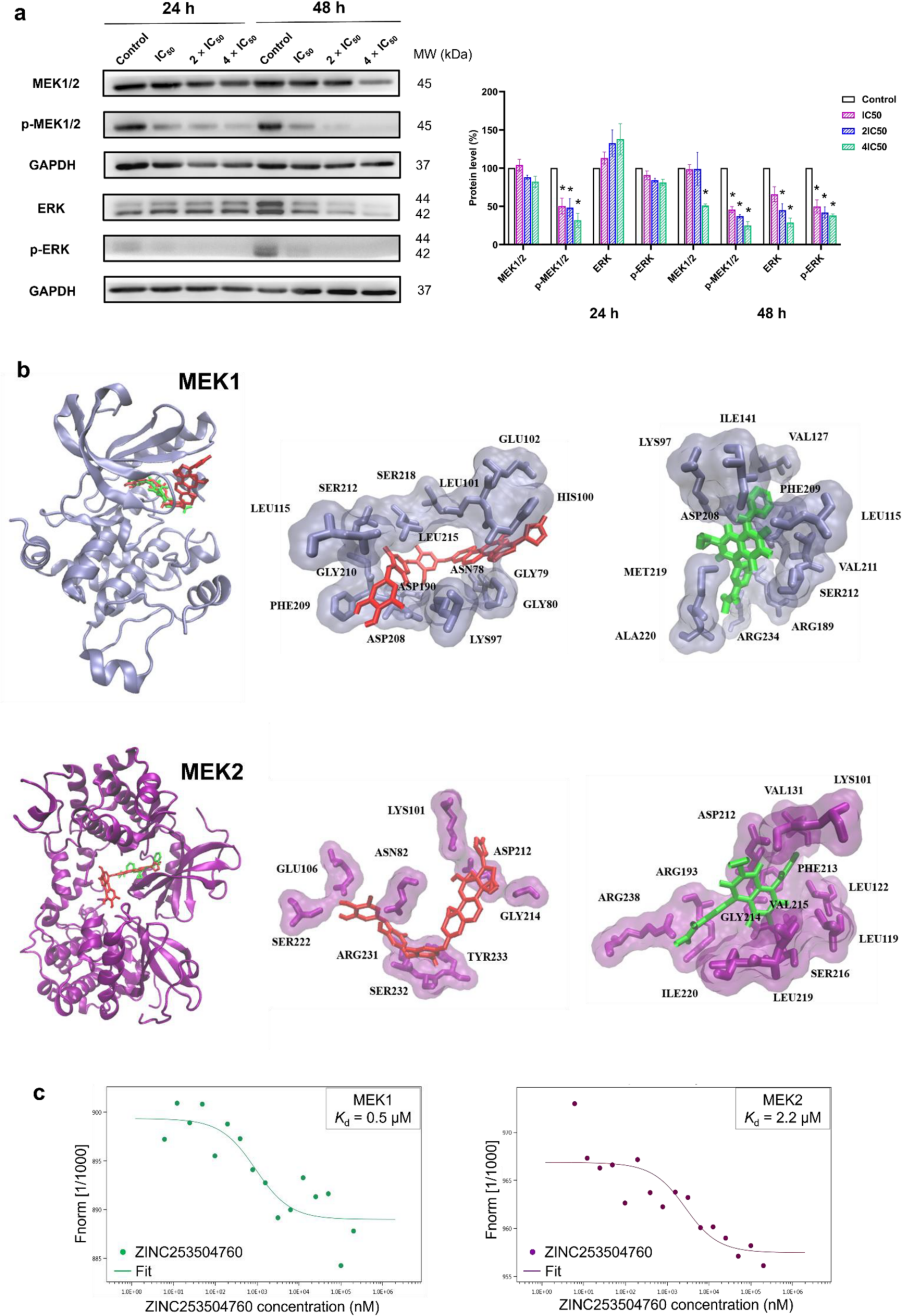
Inhibition of MEK1/2 phosphorylation. **a** Western blotting analysis of the effect of ZINC253504760 on MAPK signaling in CCRF-CEM cells. GAPDH was used as the loading control. Digitalized graphs of affected protein levels are shown below. The bars represent mean values ± SEM of three independent experiments. Statistics analysis was calculated by paired student’s t-test, * *p* ≤ 0.05, ** *p* ≤ 0.001. **b** Visualization of docking results. MEK1 (PDB code:1s9j, ice-blue) and MEK2 (PBD code:1s9i, purple) are presented in a new cartoon format. Ligands are presented in bond format with different colors. ZINC253504760 (red) and trametinib (green). **c** Binding of ZINC253504760 with MEK1 and MEK2 as determined by microscale thermophoresis. Monolith^TM^ NT analysis software was used to determine the fitted *K*_d_ on MEK1 and MEK2, and to plot the ZINC253504760 fit curve

Since MEK1/2 and ERK were predicted to be downregulated and linked with G2/M cell cycle arrest in CCRF-CEM cells using IPA-based evaluation of the microarray data, we further evaluated their expression and phosphorylation status by western blotting. Upon treatment for 24 h, ZINC253504760 significantly downregulated the phosphorylation level of p-MEK1/2, while neither significant effect on MEK 1/2 nor downregulation of ERK protein expression was observed upon treatment, indicating that ZINC253504760 inhibited MEK1/2 phosphorylation (Fig. 7a). After 48 h treatment, ZINC253504760 further downregulated ERK and p-ERK through a more significant downregulation of p-MEK1/2, indicating that ZINC253504760 inhibited ERK protein expression through inhibiting p-MEK1/2. However, there were still no noticeable changes observed on MEK1/2. The significant decrease of MEK1/2 expression with 4×IC_50_ at 48 h may result from cell death. Therefore, these data suggested that ZINC253504760 inhibited the phosphorylation of MEK1/2.

### Molecular docking

To further investigate the possible interaction of ZINC253504760 with MEK1 and MEK2, molecular *in silico* docking studies were performed. Trametinib was used as a known allosteric inhibitor of MEK1/2 to compare the binding affinities with those of ZINC253504760 (Roskoski 2017). ZINC253504760 showed binding affinity (lowest binding energy) to MEK1 (-8.15 ± 0.3 kcal/mol) and MEK2 (-7.85 ± 0.4 kcal/mol), which were approximately the same as trametinib (Supplementary Table S5). As shown in Fig. 7b, five amino acid residues of MEK1 appeared to interact with both ZINC253504760 and trametinib, *i.e*., LYS97, LEU115, ASP208, PHE209, and SER212. Remarkably, ZINC253504760 bound to SER218, which is one of the important phosphorylation sites on MEK1. Regarding MEK2 (chain B), LYS101, ASP212, and GLY214 interacted with both ZINC253504760 and trametinib. Similarly, ZINC253504760 bound to another important phosphorylation site on MEK2, SER222. Therefore, we conclude that ZINC253504760 is an ATP-competitive inhibitor and hereby affects MEK1 and MEK2 phosphorylation. Furthermore, the binding affinity of the deglycosylated form of ZINC253504760 to MEK1 was -6.26 ± 0.05 kcal/mol, and to MEK2 was -6.57 ± 0.005 kcal/mol (Supplementary Table S5), which were closed to the glycosylated form of ZINC253504760. Even though the interacting amino acids of deglycosylated form did not dock to phosphorylated sites on MEK1 or MEK2, they were at similar sites compared with trametinib.

### Detection of MEK1/2-compound binding by microscale thermophoresis

The *in vitro* interaction of ZINC253504760 with MEK1/2 was confirmed by MST (Fig. 7c). The measured concentration-dependent fluorescence signals indicated an interaction between the fluorescently labeled MEK1 and MEK2 protein with the compound. ZINC253504760 bound to MEK1 with a *K*_d_ value of 0.5 µM and to MEK2 with a *K*_d_ value of 2.2 µM.

## Discussion

Natural products have been regarded as a ‘‘treasure box’’, which greatly contributed to pharmacological research and drug development (Newman and Cragg 2020). Cardiac glycosides (CGs) are one of the leading naturally derived classes of anticancer drug candidates (Kumar and Jaitak 2019). To further develop cardenolides, the aim of the present study was to explore the molecular mode of action of a cardenolide, ZINC253504760. This is a synthetic CG compound with structural similarity to digitoxin and digoxin. Digitoxin and digoxin have been discussed as new anticancer agents (El-Seedi et al. 2019b; Menger et al. 2013), indicating the likelihood that ZINC253504760 might also show anticancer activity.

We demonstrated that this compound was not involved in the major mechanism of resistance. It is worth to point out that cross-resistance to ZINC253504760 was only observed in BCRP-overexpressing cells, multidrug resistance (except BCRP), making it attractive for further investigations. Previously, we examined the cytotoxicity of a library of 66 CGs that inhibited the efflux function of P-glycoprotein and overcame MDR (Zeino et al. 2015b).

As a next step, we selected drug-sensitive CCRF-CEM leukemia cells as model to unravel the modes of action, since the resazurin assay revealed that ZINC253504760 showed the lowest nanomolar concentration against CCRF-CEM cells. In contrast, the viabilities in embryonic kidney cells (HEK293 and HEK293/ABCB5) and breast cancer cells (MDA-MB-231-pcDNA3) were still more than 20% treated with 100 µM of ZINC253504760, which clearly means these cells lines did not exhibit significant lethality to ZINC253504760. While in glioblastoma multiform cells (U87MG and U87MGEGFR), the viabilities already decreased to 80% at 0.003 µM, which means ZINC253504760 may begin to kill cells in lower concentrations, while its IC_50_ value was the second only to leukemia cells. Leukemia remains a malignancy in children and adults due to the frequent relapses after treatment (Siegel et al. 2022). A variety of studies reported that CGs including digitoxin, bufalin, ouabain, and pervoside, which showed already profound cytotoxicity in leukemia cells (Masuda et al. 1995; Jing et al. 1994; Feng et al. 2016; Zeino et al. 2015a). Importantly, several CGs indicated that the execution of apoptosis in leukemia cells and did not affect normal blood cells, suggesting that CGs may possess at least some specific tumor-specificity (Ayogu and Odoh 2020).

Cell death was initially categorized into three types: type I (apoptosis), type II (autophagy) and type III (necrosis). Meanwhile, novel cell death modes have been attracting attention for intervention in disease mechanisms. PARP is activated by DNA strand nicks and breaks and takes part in different pathways of DNA damage repair. Different from mild DNA damage that stimulates PARP to repair damage, parthanatos is uniquely induced by severe DNA damage. It is important to point out that PARP activation is required for AIF translocation. AIF failed to translocate into the nucleus in PARP-knockout fibroblasts after MNNG treatment (Yu et al. 2002). Furthermore, PAR has been identified as an AIF-releasing factor. AIF activity was abrogated by PAR glycohydrolase, an enzyme that plays an important role in the degradation of PAR (Yu et al. 2006). Our western blot analysis revealed elevated full length-PARP (116 kDa) and increased PAR expression in a concentration-dependent manner by ZINC253504760, following decreased cytoplasmic but increased nuclear AIF expression. AIF translocation represents a key event in response to PARP-mediated cell death (Susin et al. 1999). Immunofluorescence microscopy showed that AIF indeed accumulated in the nucleus of treated cells. Currently, the mechanism of AIF release can be explained in two ways: (1) PAR translocates to the cytoplasm, interacts with mitochondria, and binds to AIF (Wang et al. 2011). (2) PAR formation consumes the NAD^+^ stores, which leads to a disruption of the MMP and AIF release (Andrabi et al. 2006). Mitochondria act as death centers under cellular stress conditions to release apoptogenic factors including AIF and cytochrome *c* (Andrabi et al. 2008). In our study, flow cytometric analyses showed that ZINC253504760-treated cells lost their MMP in a concentration-dependent manner. Therefore, our experiments support the view that AIF translocated to the nucleus because of the massive PAR accumulation and the loss of the MMP. The phosphorylation of the histone variant H2A.X represents an immediate response to DNA double-strand breaks (DSB) and has been widely applied for the detection of DNA damage (Rahmanian et al. 2021). Our data showed the p-histone H2A.X levels rose instantly upon treatment with increasing drug concentrations. To further validate this result, DNA damage was also investigated by the alkaline comet assay, which indeed revealed increased percentages of tail DNA induced by ZINC253504760. Taken together, from the key features of parthanatos, we demonstrated that ZINC253504760 resulted in rapid PARP activation, PAR accumulation, mitochondrial depolarization, AIF translocation, and large-scale DNA fragmentation. These data are consistent with the other reports where parthanatos was induced in cancer cells (Zhao et al. 2015; Ma et al. 2016). Parthanatos is the major mode of cell death of ZINC253504760 in CCRF-CEM leukemia cells. It contributes to the growth inhibition of ZINC253504760 in the resazurin assay and explains the appearance of the cell death and survival pathway predicted by the microarray-based Ingenuity Pathway Analysis.

Even if parthanatos is different from other modes of cell death regarding their morphological and molecular pathways, they interact with each other and exert cross-talks. Firstly, parthanatos may interact with apoptosis. Early studies have been demonstrated that PARP activation and AIF activity can be maintained in the presence of wide-ranging caspase inhibitor Z-VAD-fmk in MNNG treated wild-type fibroblasts, suggesting that pathanatos is a caspase-independent cell death mode (Susin et al. 1999; Yu et al. 2002), while recent emerging evidence indicates that caspases also can be activated in parthanatos (Yu et al. 2002). In our study, the PARP fragment (89 kDa) and caspase 3 participated in ZINC253504760-induced cell death. PARP is a switch point that directs cell death toward either apoptosis or parthanatos. Parthanatos starts in the overactivation of PARP and results in the deletion of energy stores, while apoptosis is initiated by cleaved PARP with abundant energy (Zhou et al. 2021). Hence, the switch of PARP might be cross-dynamic. Meanwhile, we observed the expression of cleaved PARP without cleaved caspase 3. In fact, it has been reported that PARP cleavage was not defective in the absence of caspase 3 (Slee et al. 2001). Our results supported this study. Since we did not find significant late apoptosis of ZINC253504760 treatment, we propose here, ZINC253504760 induced parthanatos as the major mode of cell death and with a minor role of apoptosis in leukemia cells. Furthermore, AIF may interact with caspase. As mentioned above, both AIF and cytochrome *c* are released from mitochondria. Cytochrome *c* redistributes into the cytosol and triggers the activation of caspase 3 or caspase 9. Kinetic studies revealed the order of action that the mitochondrial release of AIF occurred earlier than that of cytochrome c (Daugas et al. 2000b). This means that AIF release initiates stage I of chromatin condensation (caspase-independent), and in stage II cell death relies on the activation of caspase by cytochrome *c* (caspase-dependent) (Daugas et al. 2000a). Along this line, we assume that upon treatment with ZINC253504760 for 24 h (stage I), parthanatos is the predominant mode of cell death, when AIF translocates into the nucleus. In stage II (after 24 h or more), caspase 3 was gradually activated, while a small fraction of apoptosis was induced in dying cells. Furthermore, ZINC253504760 did not induce ROS-dependent parthanatos or mitochondrial apoptosis in CCRF-CEM cells, this finding is different with parthanatos induced in glioma cells (Ma et al. 2016). Finally, autophagy biomarkers including Beclin and p62 were decreased in a time- and concentration-dependent manner, suggesting that ZINC253504760 did not induce autophagy in CCRF-CEM cells. Thus, ZINC253504760-induced parthanatos did not correlate to autophagy. Our finding is consistent with parthanatos induced in esophageal cancer (Zhao et al. 2015).

Using flow cytometry, we observed that ZINC253504760 induced G2/M phase arrest, and this result was consistent with the microarray-based pathway prediction. We also further verified a number of genes involved in the cell cycle using qRT-PCR. G2/M phase arrest is one of the main cellular responses to DNA damage that prevents the transmission of damaged DNA to undergo mitosis without repair of DNA lesions. It is worth to mention that ATM (ataxia-telangiectasia mutated) and ATR (ataxia-telangiectasia mutated and Rad3-related) kinase are the required sensors to recognize DNA damage and to phosphorylate their target proteins, such as p53 and H2AX (Sancar et al. 2004). In the present study, HIPK2, a damage-activated checkpoint kinase that is activated by ATM was upregulated after treatment (Hofmann et al. 2013), indicating that ZINC253504760 induced DNA damage. PPM1D, a p53-induced protein after chemical stimulation, deactivates p53 by dephosphorylating ATM/ATR following DNA damage. Therefore, PPM1D has been categorized as an oncogene, since it suppresses the DNA repair function (Lu et al. 2004, 2005). This may explain our results that PPM1D was upregulated while p53 was downregulated. CDK7 along with cyclin H comprises the CDK-activating kinase (CAK), which is necessary to activate CDKs by providing the T-loop phosphorylation (Sava et al. 2020). In our experiment, the downregulation of CDK7 by ZINC253504760 during G2 phase actually disrupts cyclin B1-CDK1 assembling and blocks cells to enter mitosis (Larochelle et al. 2007). However, a connection between CDK7 and p53 was not predicted by our microarray data. CDK7 and p53 have been raised questions because they both share functional similarities regarding cell cycle regulation, transcription, and DNA repair. P53 can be phosphorylated by CDK7-Cyclin H in a p36^*MAT1*^-dependent manner both *in vitro* and *in vivo* (Ko et al. 1997). Hence, it is understandable in our study that CDK7 was downregulated if p53 was also downregulated. Moreover, in our study, CDK1 was upregulated both in microarray and qRT-PCR experiments, which seems to be conflicting with G2/M phase arrest at first sight. In fact, even though the cyclin B1-CDK1 complex is inactive in the G2/M phase, it still can be activated at the start of prophase, and cyclin B1-CDK1 activity reaches its maximum shortly after the nuclear envelope breakdown (Gavet and Pines 2010). This period maintains the cells in their mitotic state. Therefore, we assume that our experiments at 24 h captured this mitotic moment of CDK1 upregulation. On the other hand, cyclinB1-CDK1 was inactivated after cyclin B1 degradation in arrested cells (Porter and Donoghue 2003). The change from the active to the inactive cyclinB1-CDK1 complex has been described as hysteresis (Pomerening et al. 2003). Regarding our results, this may be another reason that CDK7 was already downregulated while CDK1 was still upregulated and active. CKS1 and CKS2 are proposed to physically link with cyclinB1-CDK1 for further phosphorylation to their substrates (Ellederova et al. 2019). The upregulation of CKS1 and CKS2 was consistent with the upregulation of CDK1. Furthermore, Wee1 is a kinase which negatively regulates CDK1 by catalyzing the phosphorylation on Thr14 and Tyr15 and which inhibits CDK1 activity (Du et al. 2020). Our data showed that Wee1 was upregulated by ZINC253504760, while CDK1 remained upregulated under hysteresis and inactivated cyclin B1-CDK1. Furthermore, the ATR-CHEK1-CDC25 pathway was predicted by IPA to be affected by ZINC253504760. This is a classical pathway activated following DNA damage and arrest in the G2 phase (Calonge and O'Connell 2008). Due to our limited treatment time (24 h) and concentration (IC_50_), this pathway was only predicted by IPA software without expression of fold change, but our validation by qRT-PCR of the eight genes is sufficient to support cell cycle G2/M arrest induced by ZINC253504760.

Microtubules have multiple functions in cellular processes, especially regarding the formation of mitotic spindles during cell cycle, making them vital therapeutic targets in cancer treatment (Dumontet and Jordan 2010). The microtubule-targeting antimitotic drugs can be divided into microtubule-destabilizing agents (*e.g.*, *Vinca* alkaloids and colchicine), and microtubule-stabilizing agents (*e.g.*, taxanes). Both drug classes block mitosis (Wang et al. 2016). We found that ZINC253504760 interfered with tubulin polymerization in U2OS cells expressing an α-tubulin-GFP construct. In brief, our data obtained from flow cytometry, qRT-PCR, and fluorescence microscopy of the microtubule cytoskeleton supported the view that ZINC253504760 induced G2/M phase arrest and blocked the cellular entry into mitosis.

A wide of human tumors are under the control of MAPK pathway for growth and survival (Sebolt-Leopold and Herrera 2004). Since the downregulation of MEK1/2 and ERK was associated with G2/M cell cycle arrest biomarkers as predicted by IPA, we confirmed that ZINC253504760 indeed resulted in G2/M phase arrest. Therefore, we also investigated the hypothesis that ZINC253504760 can downregulate MEK1/2 *in vitro*, supposing that MEK1/2 might be a target of ZINC253504760 in CCRF-CEM cells. Indeed, our study revealed that p-MEK1/2 was downregulated in a concentration- and time-dependent manner, which further affected p-ERK and ERK. While ERK was still upregulated after 24 h, MEK1/2 dephosphorylation was still ongoing and ERK was downregulated after 48 h. Next, we carried out molecular docking to understand the mode of binding of ZINC253504760 to MEK1 and MEK2. As expected, ZINC253504760 bound to the phosphorylation sites, SER218 on MEK1 and SER222 on MEK2. In human MEK1, the substitution of either SER218 or SER222 abrogates the MEK1 activation, implying that both serines are required for phosphorylation (Zheng and Guan 1994). Our results supported this finding. Therefore, we conclude that ZINC253504760 contributed to MEK1/2 inactivation, which further led to downstream ERK downregulation and inhibition of cell proliferation. In addition, the lowest binding energy of deglycosylated form of ZINC253504760 to MEK1/2 was similar to that of glycosylated form. Regarding the fact that the bioactivity of GCs decreases with the loss of sugar, the monitor of bioactive metabolites of GCs needs to be studied in the future, and if necessary, concomitant administration such as metabolic enzyme inhibitors or other drugs (*e.g.,* quinidine increased absorption of digoxin) might be options to increase CGs bioavailability (Pedersen et al. 1983; Jortani and Valdes 1997). Moreover, the ATP-binding pocket on kinases has been classified into many types (Pan and Mader 2022). MEK1/2 is one of the most thoroughly investigated kinases for type III allosteric inhibitors. The four FDA-approved MEK1/2 are all ATP-noncompetitive kinase inhibitors (Roskoski 2017; Lu et al. 2020). Our results showed trametinib as a known allosteric inhibitor bound adjacent to the ATP binding site which supported previous studies and proved that our molecular docking approach was correct. Finally, the molecular interactions of ZINC253504760 with MEK1/2 have been confirmed by MST, and the fit curves proved once again that ZINC253504760 could bind to MEK1 and MEK2. Taken together, our *in vitro* and *in silico* results indicated that ZINC253504760 bound to MEK1/2 and inhibited MEK1/2 phosphorylation.

Interestingly, numerous studies revealed the characteristic molecular mechanisms of CGs in cancer cells. First and foremost, apoptotic cell death has been described in most cases (e.g., UNBS1450) (Juncker et al. 2011). Immunogenetic cell death was also induced by CGs (*e.g*., oleandrin) (Li et al. 2021; Menger et al. 2012). In the present investigation, ZINC253504760 predominantly induced parthanatic cell death rather than apoptosis. Our finding opened a new door for further studies on the cell death mode of CGs and pointed to the potential of ZINC253504760 to treat anti-apoptosis- and drug-resistant cancers. Moreover, ZINC253504760 arrested CCRF-CEM leukemia cells in the G2/M phase of the cell cycle. This result is comparable to reports from many other CGs, such as proscillaridin A-treated glioblastoma cells (Denicolaï et al. 2014), ouabain-treated melanoma cells (Wang et al. 2021), and lanatoside C-treated breast, lung, or liver cancer cells (Reddy et al. 2019). Based on this fact, the induction of parthanatos and G2/M phase arrest in cell cycle are the best evidence to support that ZINC253504760 leads to DNA damage. In addition, Src is a non-receptor protein tyrosine kinase, the activation of the Src-EGFR-MAPK pathway is one of the accepted potential mechanisms of the anticancer effects of CGs (Prassas and Diamandis 2008; Kometiani et al. 2005). Our functional network identified from the transcriptomic analysis (Fig. 3c) showed that c-Src protein, as well as the top affected genes *Hsp90AA1* and *Hsp90 AB1* were downregulated after ZINC253504760 treatment. *Luo et al* have demonstrated that c-Src was weakly affected by the chaperone Hsp90 (Luo et al. 2017). Therefore, our study revealed the molecular mode of action of ZINC253504760 resulting from DNA damage, parthanatic cell death and G2/M arrest.

Last but not least, CG compounds have a narrow therapeutic window and show different toxicities. In the future, the selectivity and toxicity of ZINC253504760 should be investigated in more detail. In the light of developing CG compounds for the treatment of malignant diseases, studies based on pharmacokinetic properties, toxicological mechanisms and further structural modifications should be conducted to achieve reduced toxicity of CG compounds. Personalized medicine by investigating gene polymorphisms is also a strategy to delineate applicable populations with increased efficacy and less toxicity (Zhai et al. 2022).

## Conclusion

Taken together, in this study we described the mechanism of a new synthetic cardenolide compound, ZINC253504760. This compound displayed cytotoxicity against different multidrug-resistant cell lines with overexpression of ABC transporters (P-glycoprotein, ABCB5), overexpression of the activated oncogene ∆EGFR, or a knock-out of the tumor suppressor TP53. Starting from microarray-based mRNA expression profiling, we demonstrated that ZINC253504760 induced parthanatos-type cell death and G2/M phase arrest in CCRF-CEM cells, both resulting from DNA damage. ZINC253504760 induced the overexpression of PARP and PAR and nuclear AIF translocation, and disrupted the mitochondrial membrane potential, all of which are steps triggering parthanatos. Furthermore, ZINC253504760 blocked the MAPK pathway by inhibiting MEK1/2 phosphorylation in a time- and concentration-dependent manner. ZINC253504760 bound to MEK1 and MEK2 at their phosphorylated sites, as demonstrated by microscale thermophoresis and molecular docking. To the best of our knowledge, parthanatos was shown for the first time to be induced by a cardenolide compound. These results provide a basis for further exploring ZINC253504760 as an alternative strategy to treat cancer cells that have the ability to escape apoptosis and are drug-resistant. Further assessments of ZINC253504760 are warranted regarding its toxicity.

### Supplementary Information

Below is the link to the electronic supplementary material.

Supplementary file1 (DOCX 256 56KB)

## Data Availability

The authors declare that the data supporting the findings of this study are available within the paper. All other data are available from the corresponding author upon reasonable request.

## Abbreviations

AIF: apoptosis-inducing factor
ATM: ataxia-telangiectasia mutated
ATR: ataxia-telangiectasia mutated and Rad3-related
BCRP: breast cancer resistance protein
BSA: bovine serum albumin
CDK1: cyclin-dependent kinase 1
CGs: cardiac glycosides
DAPI: 4’6-diamidino-2-phenylindole
DSB: double-strand break
ERK: extracellular signal-regulated kinase
IPA: Ingenuity Pathway Analysis
JC-1: 5,5’6,6’-trtrachloro-1,1’3,3’-tetraethylbenyimidazolylcarbocyanine iodide
*K*_d_: dissociation constant
MAPK: mitogen-activated protein kinase
MDR: multidrug resistance
MEK: mitogen-activated protein kinase kinase
MEKi: MEK inhibitors
MMP: mitochondrial membrane potential
MNNG: N-methyl-N’nitro-N-nitrosoguanidine
MST: microscale thermophoresis
NAD: nicotinamide adenine dinucleotide
PAR: poly(ADP-ribose)
PARP1: poly(ADP-ribose) polymerase 1
PBS: phosphate-buffer saline
PI: propidium iodide
PS: phosphatidylserine
qRT-PCR: quantitative reverse transcription PCR
ROS: reactive oxygen species
