# Supplementary material for: The cardiac glycoside ZINC253504760 induces parthanatos-type cell death and G2/M arrest via downregulation of MEK1/2 phosphorylation in leukemia cells
Source: Cell Biol Toxicol. 2023 Jun 16;39(6):2971–97. doi: 10.1007/s10565-023-09813-w (PMC10693532; doi:10.1007/s10565-023-09813-w)
Supplement: Supplementary file 1 — Supplementary file1 (DOCX 256 56KB) [file 10565_2023_9813_MOESM1_ESM.docx]

**Supplementary file**

**The cardiac glycoside ZINC253504760 induces parthanatos-type cell death and G2/M arrest via downregulation of MEK1/2 phosphorylation in leukemia cells**

Min Zhou^1^, Joelle C. Boulos^1^, Sabine M. Klauck^2^, Thomas Efferth^1^*

^1^ Department of Pharmaceutical Biology, Institute of Pharmaceutical and Biomedical Sciences, Johannes Gutenberg University-Mainz, Staudinger Weg 5, 55128 Mainz, Germany

^2^ Division of Cancer Genome Research, German Cancer Research Center (DKFZ), German Cancer Consortium (DKTK), National Center for Tumor Disease (NCT), 69120 Heidelberg, Germany

*** Correspondence to:** Prof. Dr. Thomas Efferth, E-mail: efferth@uni-mainz.de

**Supplementary Table S1:** The sequence of primers (5´→3´) designed for qRT-PCR

|  | **Gene** | **Forward primer** | **Reverse Primer** |
| --- | --- | --- | --- |
| Technical verification | *CD82* | GGGGGCCTATGTCTTCATCG | CTTCAGCAGCCCCAGCAG |
|  | *H2AC18* | GCAACGACGAGGAACTGAAC | CTCACTTGCCCTTTGCCTTG |
|  | *HSP90AA1* | GCTCCAAGGGTTGACATGGT | TGTAACTCATGGACGCAGGG |
|  | *HSP90AB1* | TTTTAGATGCCTGAGGAAGTGC | TGAAAGGCAAAAGTCTCCACC |
| Biological verification | *HIPK2* | CCCCGTGTACGAAGGTATGG | TGAAGGGTGTGAGGGGAGAA |
|  | *PPM1D* | GCCCCCTGATGAAGAAGCAT | CTGTGAGGTTGTGGGGAGAC |
|  | *CDK1* | GGAAATTGAGCGGAGAGCGA | GGGTATGGTAGATCCCGGC |
|  | *Wee1* | CTTCCTGAGCCGACAGCAG | TGCAGGGCGAGAAGATCAG |
|  | *CKS1* | CTTGGCAGATGGAGGAAGCA | AATCCGCAAGTCACCACACA |
|  | *CKS2* | GTTAGTCTCCGGCGAGTTGT | CAGCAGACAAAACCACGTCC |
|  | *TP53* | ACCTATGGAAACTACTTCCTGAAA | TCCGGGGACAGCATCAAATC |
|  | *CDK7* | GGAACGCCAACCGCCT | CGTCGCTTTAGTATCCCCGC |
| Internal control | *GAPDH* | ATGAATGGGCAGCCGTTAGG | AGCATCACCCGGAGGAGAAA |

**Supplementary Table S2:** Downregulated genes affected with ZINC253504760 treated at a concentration of IC_50_ for 24 h in CCRF-CEM leukemia cells

| **Symbol** | **Entrez Gene Name** | **Expr Fold Change** |
| --- | --- | --- |
| HSP90AA1 | heat shock protein 90 alpha family class A member 1 | -2254.374 |
| HSP90AB1 | heat shock protein 90 alpha family class B member 1 | -2235.608 |
| ANXA2 | annexin A2 | -2214.464 |
| FDFT1 | farnesyl-diphosphate farnesyltransferase 1 | -2029.449 |
| OAZ1 | ornithine decarboxylase antizyme 1 | -1673.232 |
| MSMO1 | methylsterol monooxygenase 1 | -1629.193 |
| HMGCS1 | 3-hydroxy-3-methylglutaryl-CoA synthase 1 | -1549.102 |
| ACTG1 | actin gamma 1 | -1530.589 |
| TCP1 | t-complex 1 | -1308.835 |
| AHSA1 | activator of HSP90 ATPase activity 1 | -1241.763 |
| STIP1 | stress induced phosphoprotein 1 | -1215.613 |
| EIF2S3 | eukaryotic translation initiation factor 2 subunit gamma | -1167.775 |
| CCT4 | chaperonin containing TCP1 subunit 4 | -1160.239 |
| HNRNPM | heterogeneous nuclear ribonucleoprotein M | -1147.657 |
| ACLY | ATP citrate lyase | -1071.058 |
| DHCR7 | 7-dehydrocholesterol reductase | -1066.943 |
| INSIG1 | insulin induced gene 1 | -1063.913 |
| CACYBP | calcyclin binding protein | -1063.799 |
| LCP1 | lymphocyte cytosolic protein 1 | -993.11 |
| ATP5MF | ATP synthase membrane subunit f | -979.524 |
| CCT8 | chaperonin containing TCP1 subunit 8 | -900.789 |
| PRMT1 | protein arginine methyltransferase 1 | -868.785 |
| SCD | stearoyl-CoA desaturase | -866.407 |
| C6orf62 | chromosome 6 open reading frame 62 | -858.048 |
| FKBP4 | FKBP prolyl isomerase 4 | -856.757 |
| SLIRP | SRA stem-loop interacting RNA binding protein | -851.18 |
| ARRB2 | arrestin beta 2 | -835.199 |
| HSPB1 | heat shock protein family B (small) member 1 | -811.308 |
| CCT6A | chaperonin containing TCP1 subunit 6A | -806.584 |
| STARD7 | StAR related lipid transfer domain containing 7 | -787.833 |
| PAPOLA | poly(A) polymerase alpha | -778.453 |
| PRDX1 | peroxiredoxin 1 | -777.987 |
| NSA2 | NSA2 ribosome biogenesis factor | -737.527 |
| NCL | nucleolin | -734.727 |
| PSMD11 | proteasome 26S subunit, non-ATPase 11 | -670.18 |
| FDPS | farnesyl diphosphate synthase | -670.069 |
| CANX | calnexin | -653.05 |
| FBL | fibrillarin | -647.532 |
| PSMB8 | proteasome 20S subunit beta 8 | -638.806 |
| EIF3C | eukaryotic translation initiation factor 3 subunit C | -638.628 |
| EIF3M | eukaryotic translation initiation factor 3 subunit M | -624.461 |
| RPL34 | ribosomal protein L34 | -621.373 |
| DARS1 | aspartyl-tRNA synthetase 1 | -609.431 |
| ST13 | ST13 Hsp70 interacting protein | -605.904 |
| AIF1 | allograft inflammatory factor 1 | -605.078 |
| UCP2 | uncoupling protein 2 | -604.331 |
| PSMD1 | proteasome 26S subunit, non-ATPase 1 | -603.632 |
| PRMT5 | protein arginine methyltransferase 5 | -592.782 |
| RSL1D1 | ribosomal L1 domain containing 1 | -578.905 |
| SNU13 | small nuclear ribonucleoprotein 13 | -578.016 |
| IPO5 | importin 5 | -562.492 |
| ATP5F1E | ATP synthase F1 subunit epsilon | -562.345 |
| NAA15 | N-alpha-acetyltransferase 15, NatA auxiliary subunit | -560.327 |
| IFI16 | interferon gamma inducible protein 16 | -555.729 |
| PFKP | phosphofructokinase, platelet | -554.895 |
| UBL7 | ubiquitin like 7 | -553.865 |
| ATIC | 5-aminoimidazole-4-carboxamide ribonucleotide formyltransferase/IMP cyclohydrolase | -536.155 |
| DNAJC7 | DnaJ heat shock protein family (Hsp40) member C7 | -534.446 |
| VDAC1 | voltage dependent anion channel 1 | -519.907 |
| ANAPC5 | anaphase promoting complex subunit 5 | -516.528 |
| MTMR4 | myotubularin related protein 4 | -511.685 |
| SORD | sorbitol dehydrogenase | -502.115 |
| NDUFB4 | NADH:ubiquinone oxidoreductase subunit B4 | -496.969 |
| PSMD3 | proteasome 26S subunit, non-ATPase 3 | -493.703 |
| U2AF1/U2AF1L5 | U2 small nuclear RNA auxiliary factor 1 | -488.513 |
| ARHGDIA | Rho GDP dissociation inhibitor alpha | -488.035 |
| BZW2 | basic leucine zipper and W2 domains 2 | -486.368 |
| TOMM40 | translocase of outer mitochondrial membrane 40 | -476.07 |
| ME2 | malic enzyme 2 | -471.544 |
| DDX21 | DExD-box helicase 21 | -467.445 |
| VPS29 | VPS29 retromer complex component | -467.408 |
| ACAT2 | acetyl-CoA acetyltransferase 2 | -449.953 |
| MLLT11 | MLLT11 transcription factor 7 cofactor | -446.862 |
| HSPA4 | heat shock protein family A (Hsp70) member 4 | -445.264 |
| SF3B3 | splicing factor 3b subunit 3 | -443.602 |
| DNPEP | aspartyl aminopeptidase | -443.194 |
| DFFA | DNA fragmentation factor subunit alpha | -439.313 |
| CCNC | cyclin C | -437.182 |
| PEBP1 | phosphatidylethanolamine binding protein 1 | -436.582 |
| MRPL36 | mitochondrial ribosomal protein L36 | -435.296 |
| PGD | phosphogluconate dehydrogenase | -434.558 |
| SRSF3 | serine and arginine rich splicing factor 3 | -434.516 |
| ADRM1 | adhesion regulating molecule 1 | -434.475 |
| SNRPE | small nuclear ribonucleoprotein polypeptide E | -432.222 |
| WDR77 | WD repeat domain 77 | -426.453 |
| ZCRB1 | zinc finger CCHC-type and RNA binding motif containing 1 | -423.009 |
| LYAR | Ly1 antibody reactive | -419.51 |
| CTNNA1 | catenin alpha 1 | -418.387 |
| URI1 | URI1 prefoldin like chaperone | -417.738 |
| MATR3 | matrin 3 | -413.21 |
| DBN1 | drebrin 1 | -411.654 |
| CDC37 | cell division cycle 37, HSP90 cochaperone | -407.566 |
| CSNK1E | casein kinase 1 epsilon | -407.356 |
| LAP3 | leucine aminopeptidase 3 | -404.673 |
| PSMB7 | proteasome 20S subunit beta 7 | -404.398 |
| SEH1L | SEH1 like nucleoporin | -401.587 |
| NOLC1 | nucleolar and coiled-body phosphoprotein 1 | -384.974 |
| RNPS1 | RNA binding protein with serine rich domain 1 | -380.273 |
| APEH | acylaminoacyl-peptide hydrolase | -375.643 |
| IDI1 | isopentenyl-diphosphate delta isomerase 1 | -375.219 |
| MRPS15 | mitochondrial ribosomal protein S15 | -367.806 |
| PSMC4 | proteasome 26S subunit, ATPase 4 | -367.55 |
| ACAT1 | acetyl-CoA acetyltransferase 1 | -367.407 |
| GFUS | GDP-L-fucose synthase | -365.327 |
| MCCC2 | methylcrotonoyl-CoA carboxylase 2 | -363.918 |
| PWP1 | PWP1 homolog, endonuclein | -362.039 |
| TRIM25 | tripartite motif containing 25 | -361.951 |
| FAM162A | family with sequence similarity 162 member A | -361.051 |
| PTPN11 | protein tyrosine phosphatase non-receptor type 11 | -359.197 |
| ECD | ecdysoneless cell cycle regulator | -357.742 |
| UBE2N | ubiquitin conjugating enzyme E2 N | -353.294 |
| RPS13 | ribosomal protein S13 | -352.454 |
| NINJ2 | ninjurin 2 | -350.358 |
| AIMP2 | aminoacyl tRNA synthetase complex interacting multifunctional protein 2 | -346.993 |
| FABP5 | fatty acid binding protein 5 | -346.474 |
| UBXN4 | UBX domain protein 4 | -342.158 |
| ELOVL6 | ELOVL fatty acid elongase 6 | -340.923 |
| DNAJC2 | DnaJ heat shock protein family (Hsp40) member C2 | -339.173 |
| UBA1 | ubiquitin like modifier activating enzyme 1 | -337.431 |
| FBXW2 | F-box and WD repeat domain containing 2 | -336.769 |
| GEMIN5 | gem nuclear organelle associated protein 5 | -335.132 |
| SH3BP2 | SH3 domain binding protein 2 | -333.675 |
| PSMD14 | proteasome 26S subunit, non-ATPase 14 | -332.423 |
| INTS3 | integrator complex subunit 3 | -331.179 |
| ACOT7 | acyl-CoA thioesterase 7 | -324.602 |
| POLR3G | RNA polymerase III subunit G | -323.976 |
| METTL5 | methyltransferase like 5 | -323.449 |
| MYD88 | MYD88 innate immune signal transduction adaptor | -322.77 |
| LGALS9B | galectin 9B | -322.567 |
| BST2 | bone marrow stromal cell antigen 2 | -320.686 |
| PRPF4 | pre-mRNA processing factor 4 | -320.313 |
| POLR2L | RNA polymerase II, I and III subunit L | -319.148 |
| SLC38A5 | solute carrier family 38 member 5 | -318.882 |
| ESS2 | ess-2 splicing factor homolog | -316.132 |
| HMGCR | 3-hydroxy-3-methylglutaryl-CoA reductase | -315.911 |
| MRPL21 | mitochondrial ribosomal protein L21 | -313.143 |
| NLN | neurolysin | -312.309 |
| POLR1E | RNA polymerase I subunit E | -309.01 |
| ABLIM1 | actin binding LIM protein 1 | -308.16 |
| PMPCA | peptidase, mitochondrial processing subunit alpha | -306.64 |
| NUTF2 | nuclear transport factor 2 | -306.438 |
| UQCRH | ubiquinol-cytochrome c reductase hinge protein | -302.976 |
| SQLE | squalene epoxidase | -302.941 |
| PTDSS1 | phosphatidylserine synthase 1 | -302.924 |
| EIF2A | eukaryotic translation initiation factor 2A | -298.805 |
| THOC7 | THO complex 7 | -298.097 |
| BOLA3 | bolA family member 3 | -297.631 |
| GNL3 | G protein nucleolar 3 | -297.487 |
| IDH1 | isocitrate dehydrogenase (NADP(+)) 1 | -293.462 |
| HARS1 | histidyl-tRNA synthetase 1 | -293.128 |
| PDSS1 | decaprenyl diphosphate synthase subunit 1 | -290.247 |
| WDR46 | WD repeat domain 46 | -290.239 |
| C11orf1 | chromosome 11 open reading frame 1 | -287.56 |
| TESPA1 | thymocyte expressed, positive selection associated 1 | -286.773 |
| AASDHPPT | aminoadipate-semialdehyde dehydrogenase-phosphopantetheinyl transferase | -285.529 |
| PRORP | protein only RNase P catalytic subunit | -282.668 |
| SNRPA1 | small nuclear ribonucleoprotein polypeptide A' | -280.646 |
| EIF3H | eukaryotic translation initiation factor 3 subunit H | -279.618 |
| PABPC1 | poly(A) binding protein cytoplasmic 1 | -278.333 |
| RIOX2 | ribosomal oxygenase 2 | -276.817 |
| RRP7A | ribosomal RNA processing 7 homolog A | -275.218 |
| PRR3 | proline rich 3 | -272.484 |
| NSMCE1 | NSE1 homolog, SMC5-SMC6 complex component | -269.466 |
| GSPT1 | G1 to S phase transition 1 | -268.582 |
| MEN1 | menin 1 | -267.015 |
| VIPAS39 | VPS33B interacting protein, apical-basolateral polarity regulator, spe-39 homolog | -265.632 |
| TXNDC17 | thioredoxin domain containing 17 | -263.641 |
| THAP7 | THAP domain containing 7 | -262.941 |
| NOP16 | NOP16 nucleolar protein | -261.206 |
| MARCKSL1 | MARCKS like 1 | -260.597 |
| NKX2-5 | NK2 homeobox 5 | -257.489 |
| SMARCD1 | SWI/SNF related, matrix associated, actin dependent regulator of chromatin, subfamily d, member 1 | -255.973 |
| PPID | peptidylprolyl isomerase D | -255.409 |
| HMOX2 | heme oxygenase 2 | -254.648 |
| AADAT | aminoadipate aminotransferase | -254.49 |
| CPSF3 | cleavage and polyadenylation specific factor 3 | -248.262 |
| SMN1/SMN2 | survival of motor neuron 1, telomeric | -247.874 |
| C17orf75 | chromosome 17 open reading frame 75 | -246.645 |
| MSTO1 | misato mitochondrial distribution and morphology regulator 1 | -245.305 |
| BCL9 | BCL9 transcription coactivator | -244.657 |
| LRRC8D | leucine rich repeat containing 8 VRAC subunit D | -244.185 |
| NUDT5 | nudix hydrolase 5 | -243.537 |
| CYRIB | CYFIP related Rac1 interactor B | -243.457 |
| CYFIP2 | cytoplasmic FMR1 interacting protein 2 | -241.94 |
| MRTO4 | MRT4 homolog, ribosome maturation factor | -241.419 |
| CLTA | clathrin light chain A | -240.768 |
| DEDD | death effector domain containing | -240.718 |
| CHTF8 | chromosome transmission fidelity factor 8 | -239.237 |
| GNL2 | G protein nucleolar 2 | -238.565 |
| RRP9 | ribosomal RNA processing 9, U3 small nucleolar RNA binding protein | -237.065 |
| MCRS1 | microspherule protein 1 | -236.252 |
| MRPL2 | mitochondrial ribosomal protein L2 | -235.021 |
| KIAA1143 | KIAA1143 | -234.611 |
| TIAL1 | TIA1 cytotoxic granule associated RNA binding protein like 1 | -234.481 |
| GSR | glutathione-disulfide reductase | -230.245 |
| NUP42 | nucleoporin 42 | -228.222 |
| CCDC59 | coiled-coil domain containing 59 | -228.157 |
| PRPF6 | pre-mRNA processing factor 6 | -228.02 |
| DIMT1 | DIMT1 rRNA methyltransferase and ribosome maturation factor | -225.901 |
| IFRD1 | interferon related developmental regulator 1 | -224.319 |
| GTF2F2 | general transcription factor IIF subunit 2 | -224.286 |
| PSMA5 | proteasome 20S subunit alpha 5 | -224.113 |
| MBOAT7 | membrane bound O-acyltransferase domain containing 7 | -222.719 |
| MRPS17 | mitochondrial ribosomal protein S17 | -222.38 |
| EIF2B3 | eukaryotic translation initiation factor 2B subunit gamma | -221.69 |
| BIN2 | bridging integrator 2 | -221.242 |
| H3C1 | H3 clustered histone 1 | -221.116 |
| DDX24 | DEAD-box helicase 24 | -218.72 |
| MPI | mannose phosphate isomerase | -218.684 |
| TTLL12 | tubulin tyrosine ligase like 12 | -218.669 |
| VASH2 | vasohibin 2 | -214.566 |
| FAM86B1 | family with sequence similarity 86 member B1 | -214.041 |
| DESI1 | desumoylating isopeptidase 1 | -213 |
| ZCCHC17 | zinc finger CCHC-type containing 17 | -211.752 |
| PRIM1 | DNA primase subunit 1 | -211.63 |
| PLOD1 | procollagen-lysine,2-oxoglutarate 5-dioxygenase 1 | -210.526 |
| DDX50 | DExD-box helicase 50 | -210.452 |
| SLC39A3 | solute carrier family 39 member 3 | -210.005 |
| PM20D2 | peptidase M20 domain containing 2 | -209.237 |
| EEF1E1 | eukaryotic translation elongation factor 1 epsilon 1 | -208.556 |
| GOLGA8EP/GOLGA8G | golgin A8 family member G | -208.518 |
| PSME3IP1 | proteasome activator subunit 3 interacting protein 1 | -207.897 |
| PAFAH1B3 | platelet activating factor acetylhydrolase 1b catalytic subunit 3 | -206.402 |
| WDR18 | WD repeat domain 18 | -205.155 |
| SPRED2 | sprouty related EVH1 domain containing 2 | -204.551 |
| SETD9 | SET domain containing 9 | -203.207 |
| ACAD9 | acyl-CoA dehydrogenase family member 9 | -203.009 |
| ABRAXAS1 | abraxas 1, BRCA1 A complex subunit | -202.862 |
| TOMM22 | translocase of outer mitochondrial membrane 22 | -202.813 |
| RRP36 | ribosomal RNA processing 36 | -201.954 |
| PIR | pirin | -201.145 |
| GMPPA | GDP-mannose pyrophosphorylase A | -201.075 |
| FAM53B | family with sequence similarity 53 member B | -196.602 |
| SEPTIN2 | septin 2 | -194.014 |
| ORC3 | origin recognition complex subunit 3 | -192.886 |
| USP5 | ubiquitin specific peptidase 5 | -192.278 |
| BCAT2 | branched chain amino acid transaminase 2 | -191.816 |
| LOC283788 | FSHD region gene 1 pseudogene | -187.036 |
| UBE2D4 | ubiquitin conjugating enzyme E2 D4 (putative) | -184.849 |
| PSMD2 | proteasome 26S subunit, non-ATPase 2 | -183.511 |
| MOCS2 | molybdenum cofactor synthesis 2 | -181.781 |
| METTL2B | methyltransferase like 2B | -176.828 |
| TCEA2 | transcription elongation factor A2 | -176.394 |
| MFSD13A | major facilitator superfamily domain containing 13A | -173.922 |
| PIK3R3 | phosphoinositide-3-kinase regulatory subunit 3 | -173.38 |
| AREL1 | apoptosis resistant E3 ubiquitin protein ligase 1 | -172.377 |
| UTP18 | UTP18 small subunit processome component | -171.453 |
| MAPKAP1 | MAPK associated protein 1 | -171.117 |
| MRPS33 | mitochondrial ribosomal protein S33 | -170.341 |
| HS6ST1 | heparan sulfate 6-O-sulfotransferase 1 | -167.692 |
| RPS25 | ribosomal protein S25 | -167.496 |
| GCSH | glycine cleavage system protein H | -167.168 |
| ZNF337 | zinc finger protein 337 | -165.798 |
| APOLD1 | apolipoprotein L domain containing 1 | -165.503 |
| CMSS1 | cms1 ribosomal small subunit homolog | -163.39 |
| EHMT2 | euchromatic histone lysine methyltransferase 2 | -162.392 |
| SLC25A32 | solute carrier family 25 member 32 | -161.057 |
| CEP63 | centrosomal protein 63 | -160.992 |
| PSMA1 | proteasome 20S subunit alpha 1 | -160.829 |
| CEP104 | centrosomal protein 104 | -159.7 |
| NDUFA8 | NADH:ubiquinone oxidoreductase subunit A8 | -159.59 |
| USP9X | ubiquitin specific peptidase 9 X-linked | -158.397 |
| TFB2M | transcription factor B2, mitochondrial | -157.159 |
| TNFRSF8 | TNF receptor superfamily member 8 | -156.741 |
| ABCF2 | ATP binding cassette subfamily F member 2 | -156.465 |
| BCL11A | BAF chromatin remodeling complex subunit BCL11A | -155.916 |
| MIPEP | mitochondrial intermediate peptidase | -155.82 |
| PRX | periaxin | -155.527 |
| RAB44 | RAB44, member RAS oncogene family | -153.581 |
| MREG | melanoregulin | -153.265 |
| TRAF3IP1 | TRAF3 interacting protein 1 | -151.872 |
| ADAT1 | adenosine deaminase tRNA specific 1 | -151.718 |
| EXOSC5 | exosome component 5 | -151.668 |
| ELAC1 | elaC ribonuclease Z 1 | -151.208 |
| GBE1 | 1,4-alpha-glucan branching enzyme 1 | -151.086 |
| MPZL1 | myelin protein zero like 1 | -150.448 |
| MVD | mevalonate diphosphate decarboxylase | -149.953 |
| GTF2H3 | general transcription factor IIH subunit 3 | -149.256 |
| ABCD3 | ATP binding cassette subfamily D member 3 | -147.944 |
| SIAH2 | siah E3 ubiquitin protein ligase 2 | -146.209 |
| HPRT1 | hypoxanthine phosphoribosyltransferase 1 | -145.682 |
| TBCK | TBC1 domain containing kinase | -145.48 |
| SZRD1 | SUZ RNA binding domain containing 1 | -144.401 |
| SLC19A1 | solute carrier family 19 member 1 | -144.292 |
| GEMIN6 | gem nuclear organelle associated protein 6 | -144.049 |
| FRG1 | FSHD region gene 1 | -143.373 |
| PDCD2L | programmed cell death 2 like | -143.126 |
| VASP | vasodilator stimulated phosphoprotein | -142.505 |
| EIF2B1 | eukaryotic translation initiation factor 2B subunit alpha | -140.797 |
| JAGN1 | jagunal homolog 1 | -140.612 |
| SMARCAL1 | SWI/SNF related, matrix associated, actin dependent regulator of chromatin, subfamily a like 1 | -138.817 |
| UBA3 | ubiquitin like modifier activating enzyme 3 | -136.909 |
| WASF1 | WASP family member 1 | -136.121 |
| PIK3AP1 | phosphoinositide-3-kinase adaptor protein 1 | -135.973 |
| TRMT1L | tRNA methyltransferase 1 like | -134.415 |
| ARL10 | ADP ribosylation factor like GTPase 10 | -134.09 |
| SRRD | SRR1 domain containing | -132.293 |
| PCYOX1L | prenylcysteine oxidase 1 like | -131.234 |
| CCNH | cyclin H | -131.065 |
| PRKRA | protein activator of interferon induced protein kinase EIF2AK2 | -130.971 |
| LPCAT1 | lysophosphatidylcholine acyltransferase 1 | -130.855 |
| SLC4A11 | solute carrier family 4 member 11 | -129.678 |
| FKBP7 | FKBP prolyl isomerase 7 | -126.943 |
| TRIM59 | tripartite motif containing 59 | -126.836 |
| CPT2 | carnitine palmitoyltransferase 2 | -126.268 |
| BAG2 | BAG cochaperone 2 | -125.969 |
| FBXO9 | F-box protein 9 | -125.895 |
| SMAGP | small cell adhesion glycoprotein | -124.65 |
| ASCC1 | activating signal cointegrator 1 complex subunit 1 | -124.42 |
| GRAP2 | GRB2 related adaptor protein 2 | -123.881 |
| DEF8 | differentially expressed in FDCP 8 homolog | -122.835 |
| VARS1 | valyl-tRNA synthetase 1 | -122.509 |
| PDXP | pyridoxal phosphatase | -122.444 |
| EEFSEC | eukaryotic elongation factor, selenocysteine-tRNA specific | -122.105 |
| BIK | BCL2 interacting killer | -121.14 |
| TTF1 | transcription termination factor 1 | -120.888 |
| IVNS1ABP | influenza virus NS1A binding protein | -120.166 |
| CHAC2 | ChaC glutathione specific gamma-glutamylcyclotransferase 2 | -119.856 |
| GEMIN4 | gem nuclear organelle associated protein 4 | -119.308 |
| GART | phosphoribosylglycinamide formyltransferase, phosphoribosylglycinamide synthetase, phosphoribosylaminoimidazole synthetase | -117.878 |
| ACSL4 | acyl-CoA synthetase long chain family member 4 | -116.34 |
| PECAM1 | platelet and endothelial cell adhesion molecule 1 | -115.908 |
| EIF2B4 | eukaryotic translation initiation factor 2B subunit delta | -114.829 |
| MANF | mesencephalic astrocyte derived neurotrophic factor | -114.21 |
| SKA3 | spindle and kinetochore associated complex subunit 3 | -113.63 |
| TAB3 | TGF-beta activated kinase 1 (MAP3K7) binding protein 3 | -113.215 |
| LOC102724159/PWP2 | PWP2 small subunit processome component | -112.826 |
| PCYT2 | phosphate cytidylyltransferase 2, ethanolamine | -112.536 |
| ZNF672 | zinc finger protein 672 | -112.1 |
| NDUFC1 | NADH:ubiquinone oxidoreductase subunit C1 | -112.086 |
| PLAA | phospholipase A2 activating protein | -111.591 |
| MRS2 | magnesium transporter MRS2 | -111.473 |
| VPS18 | VPS18 core subunit of CORVET and HOPS complexes | -111.041 |
| CDK7 | cyclin dependent kinase 7 | -109.931 |
| ZNF639 | zinc finger protein 639 | -109.03 |
| COPS5 | COP9 signalosome subunit 5 | -107.352 |
| HEATR3 | HEAT repeat containing 3 | -106.675 |
| FANCF | FA complementation group F | -105.905 |
| SPRING1 | SREBF pathway regulator in golgi 1 | -105.491 |
| GLRX2 | glutaredoxin 2 | -105.145 |
| TMBIM4 | transmembrane BAX inhibitor motif containing 4 | -105.032 |
| DBNL | drebrin like | -103.451 |
| SGF29 | SAGA complex associated factor 29 | -103.158 |
| TMEM169 | transmembrane protein 169 | -103.141 |
| MZB1 | marginal zone B and B1 cell specific protein | -102.044 |
| CD52 | CD52 molecule | -101.961 |
| METAP1 | methionyl aminopeptidase 1 | -101.571 |
| BPHL | biphenyl hydrolase like | -101.131 |
| CMAS | cytidine monophosphate N-acetylneuraminic acid synthetase | -100.537 |
| TAF12 | TATA-box binding protein associated factor 12 | -100.218 |
| MCRIP2 | MAPK regulated corepressor interacting protein 2 | -99.936 |
| PRPS1 | phosphoribosyl pyrophosphate synthetase 1 | -99.895 |
| CHST2 | carbohydrate sulfotransferase 2 | -99.043 |
| ALDH5A1 | aldehyde dehydrogenase 5 family member A1 | -98.905 |
| FXN | frataxin | -97.303 |
| SFXN4 | sideroflexin 4 | -97.102 |
| NAGA | alpha-N-acetylgalactosaminidase | -95.421 |
| B4GALNT1 | beta-1,4-N-acetyl-galactosaminyltransferase 1 | -94.51 |
| CYBC1 | cytochrome b-245 chaperone 1 | -93.972 |
| HOMER3 | homer scaffold protein 3 | -92.72 |
| ELP6 | elongator acetyltransferase complex subunit 6 | -92.426 |
| BAG3 | BAG cochaperone 3 | -91.882 |
| MKNK2 | MAPK interacting serine/threonine kinase 2 | -91.236 |
| MRPS26 | mitochondrial ribosomal protein S26 | -89.42 |
| DCAF17 | DDB1 and CUL4 associated factor 17 | -88.49 |
| IARS1 | isoleucyl-tRNA synthetase 1 | -87.679 |
| TGFBRAP1 | transforming growth factor beta receptor associated protein 1 | -87.12 |
| DHX35 | DEAH-box helicase 35 | -85.433 |

**Supplementary Table S3:** Upregulated genes affected with ZINC253504760 treated at a concentration of IC_50_ for 24 h in CCRF-CEM leukemia cells

| **Symbol** | **Entrez Gene Name** | **Expr Fold Change** |
| --- | --- | --- |
| NRSN2-AS1 | NRSN2 antisense RNA 1 | 84.065 |
| PRKAR1A | protein kinase cAMP-dependent type I regulatory subunit alpha | 86.509 |
| MYO9A | myosin IXA | 88.16 |
| WIPF2 | WAS/WASL interacting protein family member 2 | 89.407 |
| PRKAB2 | protein kinase AMP-activated non-catalytic subunit beta 2 | 89.49 |
| ZNF367 | zinc finger protein 367 | 89.507 |
| ADAMTS19 | ADAM metallopeptidase with thrombospondin type 1 motif 19 | 90.115 |
| KIF15 | kinesin family member 15 | 90.142 |
| CCDC15 | coiled-coil domain containing 15 | 90.369 |
| SHLD1 | shieldin complex subunit 1 | 90.823 |
| SKA1 | spindle and kinetochore associated complex subunit 1 | 90.858 |
| IVD | isovaleryl-CoA dehydrogenase | 90.95 |
| IRAK3 | interleukin 1 receptor associated kinase 3 | 90.996 |
| MEF2D | myocyte enhancer factor 2D | 91.837 |
| FIGNL1 | fidgetin like 1 | 92.258 |
| EMC1 | ER membrane protein complex subunit 1 | 92.77 |
| TMUB2 | transmembrane and ubiquitin like domain containing 2 | 92.808 |
| ZNF684 | zinc finger protein 684 | 92.928 |
| CREBRF | CREB3 regulatory factor | 94.603 |
| ARHGEF39 | Rho guanine nucleotide exchange factor 39 | 94.946 |
| CRTC3 | CREB regulated transcription coactivator 3 | 95.144 |
| SETD1B | SET domain containing 1B, histone lysine methyltransferase | 95.24 |
| USPL1 | ubiquitin specific peptidase like 1 | 95.643 |
| CENATAC | centrosomal AT-AC splicing factor | 95.748 |
| CDC42BPA | CDC42 binding protein kinase alpha | 95.846 |
| ZBTB33 | zinc finger and BTB domain containing 33 | 96.751 |
| ARID4B | AT-rich interaction domain 4B | 96.91 |
| ELF4 | E74 like ETS transcription factor 4 | 96.96 |
| GPSM2 | G protein signaling modulator 2 | 97.484 |
| CLASP2 | cytoplasmic linker associated protein 2 | 97.876 |
| SLC2A1 | solute carrier family 2 member 1 | 98.826 |
| PHTF2 | putative homeodomain transcription factor 2 | 99.456 |
| RAD23A | RAD23 homolog A, nucleotide excision repair protein | 99.572 |
| ZHX1 | zinc fingers and homeoboxes 1 | 99.798 |
| TAF1A | TATA-box binding protein associated factor, RNA polymerase I subunit A | 100.019 |
| DHTKD1 | dehydrogenase E1 and transketolase domain containing 1 | 100.269 |
| IPMK | inositol polyphosphate multikinase | 100.3 |
| ZNF175 | zinc finger protein 175 | 100.914 |
| VCPKMT | valosin containing protein lysine methyltransferase | 101.004 |
| ZFAND4 | zinc finger AN1-type containing 4 | 101.029 |
| SLF2 | SMC5-SMC6 complex localization factor 2 | 102.071 |
| ABCA7 | ATP binding cassette subfamily A member 7 | 102.441 |
| GORAB | golgin, RAB6 interacting | 104.249 |
| SUPT6H | SPT6 homolog, histone chaperone and transcription elongation factor | 104.589 |
| REV3L | REV3 like, DNA directed polymerase zeta catalytic subunit | 104.958 |
| WDTC1 | WD and tetratricopeptide repeats 1 | 105.052 |
| RELL1 | RELT like 1 | 106.333 |
| GATD1 | glutamine amidotransferase like class 1 domain containing 1 | 106.407 |
| KCNIP3 | potassium voltage-gated channel interacting protein 3 | 107.357 |
| SPG11 | SPG11 vesicle trafficking associated, spatacsin | 107.763 |
| FOXK1 | forkhead box K1 | 108.147 |
| CHAF1A | chromatin assembly factor 1 subunit A | 108.201 |
| EXOSC10 | exosome component 10 | 108.35 |
| PBX3 | PBX homeobox 3 | 108.78 |
| ITGB3BP | integrin subunit beta 3 binding protein | 109.619 |
| VTI1A | vesicle transport through interaction with t-SNAREs 1A | 110.398 |
| CDK5RAP2 | CDK5 regulatory subunit associated protein 2 | 110.573 |
| CCDC144B | coiled-coil domain containing 144B (pseudogene) | 110.636 |
| GPATCH1 | G-patch domain containing 1 | 110.711 |
| HROB | homologous recombination factor with OB-fold | 110.833 |
| TTC7B | tetratricopeptide repeat domain 7B | 111.419 |
| ANAPC10 | anaphase promoting complex subunit 10 | 111.612 |
| TXNL4A | thioredoxin like 4A | 111.843 |
| RFXAP | regulatory factor X associated protein | 111.899 |
| PGAP6 | post-glycosylphosphatidylinositol attachment to proteins 6 | 112.608 |
| N4BP2 | NEDD4 binding protein 2 | 112.699 |
| PBK | PDZ binding kinase | 115.487 |
| DAZAP1 | DAZ associated protein 1 | 115.735 |
| REST | RE1 silencing transcription factor | 116.045 |
| ANKRD42 | ankyrin repeat domain 42 | 116.641 |
| UBR2 | ubiquitin protein ligase E3 component n-recognin 2 | 116.825 |
| VPS11 | VPS11 core subunit of CORVET and HOPS complexes | 117.681 |
| YPEL1 | yippee like 1 | 118.502 |
| ZNF571 | zinc finger protein 571 | 118.852 |
| WRN | WRN RecQ like helicase | 119.348 |
| ACVR1B | activin A receptor type 1B | 119.536 |
| LRRC37B | leucine rich repeat containing 37B | 120.01 |
| TMED3 | transmembrane p24 trafficking protein 3 | 120.698 |
| COP1 | COP1 E3 ubiquitin ligase | 120.743 |
| TMEM87B | transmembrane protein 87B | 121.818 |
| WDR19 | WD repeat domain 19 | 121.867 |
| THOC2 | THO complex 2 | 122.423 |
| ZBTB20 | zinc finger and BTB domain containing 20 | 122.932 |
| PHF19 | PHD finger protein 19 | 123.295 |
| PHF20 | PHD finger protein 20 | 123.559 |
| PDE12 | phosphodiesterase 12 | 123.99 |
| CREB1 | cAMP responsive element binding protein 1 | 124.318 |
| ZC3HAV1L | zinc finger CCCH-type containing, antiviral 1 like | 125.161 |
| BOD1L1 | biorientation of chromosomes in cell division 1 like 1 | 125.824 |
| KDM3A | lysine demethylase 3A | 125.974 |
| COPS9 | COP9 signalosome subunit 9 | 126.482 |
| SELENOS | selenoprotein S | 127.152 |
| HUS1 | HUS1 checkpoint clamp component | 127.706 |
| C10orf88 | chromosome 10 open reading frame 88 | 127.837 |
| MAD2L1 | mitotic arrest deficient 2 like 1 | 128.483 |
| BMT2 | base methyltransferase of 25S rRNA 2 homolog | 129.454 |
| CUL3 | cullin 3 | 130.174 |
| RNF121 | ring finger protein 121 | 130.49 |
| CEP95 | centrosomal protein 95 | 130.51 |
| PAPOLG | poly(A) polymerase gamma | 131.247 |
| ZNF484 | zinc finger protein 484 | 131.305 |
| INSIG2 | insulin induced gene 2 | 132.678 |
| TRAPPC2 | trafficking protein particle complex subunit 2 | 133.146 |
| RNF139 | ring finger protein 139 | 133.722 |
| BLCAP | BLCAP apoptosis inducing factor | 134.057 |
| TM6SF1 | transmembrane 6 superfamily member 1 | 134.076 |
| ITCH | itchy E3 ubiquitin protein ligase | 134.241 |
| MBD1 | methyl-CpG binding domain protein 1 | 134.306 |
| GSK3B | glycogen synthase kinase 3 beta | 134.312 |
| SETX | senataxin | 134.415 |
| ZFYVE16 | zinc finger FYVE-type containing 16 | 134.508 |
| SLF1 | SMC5-SMC6 complex localization factor 1 | 134.877 |
| NOL4L | nucleolar protein 4 like | 135.099 |
| SLC35F5 | solute carrier family 35 member F5 | 135.681 |
| GPR183 | G protein-coupled receptor 183 | 136.186 |
| ING5 | inhibitor of growth family member 5 | 136.325 |
| CDADC1 | cytidine and dCMP deaminase domain containing 1 | 136.477 |
| ZFP30 | ZFP30 zinc finger protein | 136.736 |
| KIAA2026 | KIAA2026 | 138.327 |
| ZFAND2B | zinc finger AN1-type containing 2B | 138.535 |
| TCTN2 | tectonic family member 2 | 139.143 |
| PDK2 | pyruvate dehydrogenase kinase 2 | 139.196 |
| SP4 | Sp4 transcription factor | 139.326 |
| SS18L2 | SS18 like 2 | 139.54 |
| TRIM56 | tripartite motif containing 56 | 139.834 |
| PIAS1 | protein inhibitor of activated STAT 1 | 140.645 |
| FAM200B | family with sequence similarity 200 member B | 140.965 |
| LINS1 | lines homolog 1 | 141.051 |
| BAZ2B | bromodomain adjacent to zinc finger domain 2B | 141.054 |
| CDC25A | cell division cycle 25A | 142.414 |
| MCFD2 | multiple coagulation factor deficiency 2, ER cargo receptor complex subunit | 143.348 |
| MGST2 | microsomal glutathione S-transferase 2 | 143.749 |
| DGKH | diacylglycerol kinase eta | 143.832 |
| CNTROB | centrobin, centriole duplication and spindle assembly protein | 144.191 |
| KLF12 | Kruppel like factor 12 | 145.097 |
| ERP44 | endoplasmic reticulum protein 44 | 145.421 |
| USE1 | unconventional SNARE in the ER 1 | 145.817 |
| ITPR2 | inositol 1,4,5-trisphosphate receptor type 2 | 145.87 |
| CDC45 | cell division cycle 45 | 145.901 |
| DBT | dihydrolipoamide branched chain transacylase E2 | 146.427 |
| DVL3 | dishevelled segment polarity protein 3 | 146.607 |
| HOOK3 | hook microtubule tethering protein 3 | 146.748 |
| IER3IP1 | immediate early response 3 interacting protein 1 | 149.438 |
| HERC5 | HECT and RLD domain containing E3 ubiquitin protein ligase 5 | 149.94 |
| RASSF1 | Ras association domain family member 1 | 150.068 |
| IL9R | interleukin 9 receptor | 150.417 |
| SCMH1 | Scm polycomb group protein homolog 1 | 150.605 |
| MPRIP | myosin phosphatase Rho interacting protein | 150.698 |
| TNFAIP8 | TNF alpha induced protein 8 | 151.219 |
| POLR2J2/POLR2J3 | RNA polymerase II subunit J2 | 151.486 |
| ZNF674 | zinc finger protein 674 | 151.589 |
| CCDC150 | coiled-coil domain containing 150 | 151.751 |
| TMEM86B | transmembrane protein 86B | 152.145 |
| PCMTD1 | protein-L-isoaspartate (D-aspartate) O-methyltransferase domain containing 1 | 152.79 |
| SPTLC1 | serine palmitoyltransferase long chain base subunit 1 | 152.896 |
| BSCL2 | BSCL2 lipid droplet biogenesis associated, seipin | 153.43 |
| TNFSF13B | TNF superfamily member 13b | 153.589 |
| EDEM1 | ER degradation enhancing alpha-mannosidase like protein 1 | 154.381 |
| CREBZF | CREB/ATF bZIP transcription factor | 155.666 |
| UFSP2 | UFM1 specific peptidase 2 | 155.857 |
| UBE2Z | ubiquitin conjugating enzyme E2 Z | 155.987 |
| EYA1 | EYA transcriptional coactivator and phosphatase 1 | 156.21 |
| LIMD1 | LIM domain containing 1 | 158.561 |
| RAB2B | RAB2B, member RAS oncogene family | 158.652 |
| RAPGEF6 | Rap guanine nucleotide exchange factor 6 | 158.744 |
| DCUN1D2 | defective in cullin neddylation 1 domain containing 2 | 159.457 |
| ZFP91 | ZFP91 zinc finger protein, atypical E3 ubiquitin ligase | 161.008 |
| SSH1 | slingshot protein phosphatase 1 | 161.262 |
| ZNF852 | zinc finger protein 852 | 161.32 |
| PHC1 | polyhomeotic homolog 1 | 161.832 |
| BBS10 | Bardet-Biedl syndrome 10 | 161.872 |
| SENP7 | SUMO specific peptidase 7 | 162.781 |
| ARL2BP | ADP ribosylation factor like GTPase 2 binding protein | 163.097 |
| CYP20A1 | cytochrome P450 family 20 subfamily A member 1 | 163.24 |
| PHLPP1 | PH domain and leucine rich repeat protein phosphatase 1 | 163.41 |
| UBR1 | ubiquitin protein ligase E3 component n-recognin 1 | 164.971 |
| EXOG | exo/endonuclease G | 165.192 |
| ZFX | zinc finger protein X-linked | 165.357 |
| DGKD | diacylglycerol kinase delta | 165.987 |
| MFSD14B | major facilitator superfamily domain containing 14B | 166.108 |
| RAB22A | RAB22A, member RAS oncogene family | 166.235 |
| NFATC2 | nuclear factor of activated T cells 2 | 166.647 |
| VPS45 | vacuolar protein sorting 45 homolog | 167.631 |
| LPCAT4 | lysophosphatidylcholine acyltransferase 4 | 169.316 |
| RAB3GAP1 | RAB3 GTPase activating protein catalytic subunit 1 | 170.079 |
| LYSMD3 | LysM domain containing 3 | 171.627 |
| NHLRC3 | NHL repeat containing 3 | 172.379 |
| ARID2 | AT-rich interaction domain 2 | 173.284 |
| LTN1 | listerin E3 ubiquitin protein ligase 1 | 173.56 |
| TENT5A | terminal nucleotidyltransferase 5A | 173.907 |
| ZNF654 | zinc finger protein 654 | 174.503 |
| MAPRE3 | microtubule associated protein RP/EB family member 3 | 174.745 |
| UTRN | utrophin | 175.102 |
| ANKRD17 | ankyrin repeat domain 17 | 175.15 |
| SLC25A14 | solute carrier family 25 member 14 | 176.026 |
| CDC25C | cell division cycle 25C | 176.57 |
| SOS2 | SOS Ras/Rho guanine nucleotide exchange factor 2 | 176.64 |
| WDR20 | WD repeat domain 20 | 177.567 |
| ZNF407 | zinc finger protein 407 | 178.109 |
| SCAI | suppressor of cancer cell invasion | 178.724 |
| YBEY | ybeY metalloendoribonuclease | 179.203 |
| ANKRD50 | ankyrin repeat domain 50 | 179.474 |
| PXMP4 | peroxisomal membrane protein 4 | 179.769 |
| WDR45B | WD repeat domain 45B | 180.443 |
| SLC44A5 | solute carrier family 44 member 5 | 181.007 |
| TACC3 | transforming acidic coiled-coil containing protein 3 | 181.45 |
| FAM135A | family with sequence similarity 135 member A | 181.92 |
| LEMD3 | LEM domain containing 3 | 182.23 |
| ATP1B3 | ATPase Na+/K+ transporting subunit beta 3 | 182.97 |
| SEC24A | SEC24 homolog A, COPII coat complex component | 183.273 |
| BARD1 | BRCA1 associated RING domain 1 | 184.498 |
| DAGLB | diacylglycerol lipase beta | 184.955 |
| DLL1 | delta like canonical Notch ligand 1 | 185.356 |
| VRK3 | VRK serine/threonine kinase 3 | 186.122 |
| TNRC6B | trinucleotide repeat containing adaptor 6B | 187.998 |
| RANGRF | RAN guanine nucleotide release factor | 189.488 |
| CD58 | CD58 molecule | 190.248 |
| KYAT3 | kynurenine aminotransferase 3 | 190.405 |
| CLK4 | CDC like kinase 4 | 190.508 |
| SLC30A1 | solute carrier family 30 member 1 | 190.64 |
| TGOLN2 | trans-golgi network protein 2 | 190.707 |
| DHFR | dihydrofolate reductase | 191.131 |
| C3orf38 | chromosome 3 open reading frame 38 | 192.438 |
| CDC14A | cell division cycle 14A | 193.284 |
| CEP295 | centrosomal protein 295 | 193.626 |
| REL | REL proto-oncogene, NF-kB subunit | 193.884 |
| SGMS1 | sphingomyelin synthase 1 | 194.106 |
| ERLIN1 | ER lipid raft associated 1 | 194.791 |
| USP12 | ubiquitin specific peptidase 12 | 195.191 |
| SIK2 | salt inducible kinase 2 | 196.384 |
| RICTOR | RPTOR independent companion of MTOR complex 2 | 196.621 |
| BRD8 | bromodomain containing 8 | 196.776 |
| STAM | signal transducing adaptor molecule | 197.117 |
| DCAF7 | DDB1 and CUL4 associated factor 7 | 197.755 |
| RAB14 | RAB14, member RAS oncogene family | 200.856 |
| PRCP | prolylcarboxypeptidase | 201.641 |
| MAPKAPK2 | MAPK activated protein kinase 2 | 202.499 |
| NNT | nicotinamide nucleotide transhydrogenase | 202.876 |
| ARHGAP35 | Rho GTPase activating protein 35 | 203.24 |
| YIPF3 | Yip1 domain family member 3 | 203.95 |
| HERC3 | HECT and RLD domain containing E3 ubiquitin protein ligase 3 | 204.977 |
| RBM48 | RNA binding motif protein 48 | 205.405 |
| BIRC6 | baculoviral IAP repeat containing 6 | 207.876 |
| MLXIP | MLX interacting protein | 207.903 |
| CCDC134 | coiled-coil domain containing 134 | 208.522 |
| NR2C1 | nuclear receptor subfamily 2 group C member 1 | 209.179 |
| XRN1 | 5'-3' exoribonuclease 1 | 209.415 |
| GSE1 | Gse1 coiled-coil protein | 209.517 |
| BAZ1B | bromodomain adjacent to zinc finger domain 1B | 209.739 |
| SBNO2 | strawberry notch homolog 2 | 209.976 |
| SLC12A2 | solute carrier family 12 member 2 | 210.701 |
| TMEM64 | transmembrane protein 64 | 211.597 |
| JKAMP | JNK1/MAPK8 associated membrane protein | 211.843 |
| CUX1 | cut like homeobox 1 | 212.066 |
| TET2 | tet methylcytosine dioxygenase 2 | 212.598 |
| H1-0 | H1.0 linker histone | 213.262 |
| ITSN2 | intersectin 2 | 213.266 |
| MCL1 | MCL1 apoptosis regulator, BCL2 family member | 215.094 |
| CCNL2 | cyclin L2 | 215.786 |
| NR3C1 | nuclear receptor subfamily 3 group C member 1 | 216.078 |
| FAM102B | family with sequence similarity 102 member B | 216.645 |
| FAM193A | family with sequence similarity 193 member A | 216.724 |
| CDS2 | CDP-diacylglycerol synthase 2 | 217.61 |
| TMEM41B | transmembrane protein 41B | 219.735 |
| TMEM106B | transmembrane protein 106B | 219.902 |
| NOP10 | NOP10 ribonucleoprotein | 219.969 |
| PRPSAP1 | phosphoribosyl pyrophosphate synthetase associated protein 1 | 220.242 |
| USP37 | ubiquitin specific peptidase 37 | 220.82 |
| ACO2 | aconitase 2 | 221.171 |
| ZNF721 | zinc finger protein 721 | 222.461 |
| UMAD1 | UBAP1-MVB12-associated (UMA) domain containing 1 | 222.525 |
| GGH | gamma-glutamyl hydrolase | 223.121 |
| MICOS10 | mitochondrial contact site and cristae organizing system subunit 10 | 223.173 |
| SDE2 | SDE2 telomere maintenance homolog | 223.399 |
| FRYL | FRY like transcription coactivator | 226.658 |
| WDR62 | WD repeat domain 62 | 227.145 |
| WBP4 | WW domain binding protein 4 | 227.321 |
| GOLGA7 | golgin A7 | 228.388 |
| H2AZ1 | H2A.Z variant histone 1 | 228.821 |
| SBNO1 | strawberry notch homolog 1 | 229.19 |
| IGF1R | insulin like growth factor 1 receptor | 231.74 |
| CAPRIN2 | caprin family member 2 | 233.629 |
| SLC6A9 | solute carrier family 6 member 9 | 233.972 |
| ZRANB2 | zinc finger RANBP2-type containing 2 | 234.566 |
| HECTD4 | HECT domain E3 ubiquitin protein ligase 4 | 235.145 |
| ST3GAL3 | ST3 beta-galactoside alpha-2,3-sialyltransferase 3 | 235.277 |
| ATP6V0C | ATPase H+ transporting V0 subunit c | 237.943 |
| ABHD17B | abhydrolase domain containing 17B, depalmitoylase | 238.223 |
| PIK3C2A | phosphatidylinositol-4-phosphate 3-kinase catalytic subunit type 2 alpha | 238.855 |
| RC3H2 | ring finger and CCCH-type domains 2 | 240.489 |
| ASH1L | ASH1 like histone lysine methyltransferase | 240.733 |
| CHMP3 | charged multivesicular body protein 3 | 242.248 |
| ZNF700 | zinc finger protein 700 | 242.747 |
| ITGA5 | integrin subunit alpha 5 | 243.205 |
| FNIP1 | folliculin interacting protein 1 | 243.373 |
| CIT | citron rho-interacting serine/threonine kinase | 244.515 |
| BRIP1 | BRCA1 interacting protein C-terminal helicase 1 | 246.468 |
| ZMIZ1 | zinc finger MIZ-type containing 1 | 246.481 |
| SLC16A7 | solute carrier family 16 member 7 | 248.356 |
| CHD1 | chromodomain helicase DNA binding protein 1 | 248.477 |
| RBM4 | RNA binding motif protein 4 | 249.368 |
| POLDIP3 | DNA polymerase delta interacting protein 3 | 249.603 |
| PAXBP1 | PAX3 and PAX7 binding protein 1 | 250.543 |
| PPM1D | protein phosphatase, Mg2+/Mn2+ dependent 1D | 252.506 |
| SPPL2B | signal peptide peptidase like 2B | 252.85 |
| SCLT1 | sodium channel and clathrin linker 1 | 254.669 |
| ARHGAP5 | Rho GTPase activating protein 5 | 254.813 |
| ICAM2 | intercellular adhesion molecule 2 | 257.3 |
| PPP4R3A | protein phosphatase 4 regulatory subunit 3A | 257.584 |
| BGLAP | bone gamma-carboxyglutamate protein | 257.771 |
| CNOT1 | CCR4-NOT transcription complex subunit 1 | 260.229 |
| KLHDC2 | kelch domain containing 2 | 262.514 |
| TNFRSF10C | TNF receptor superfamily member 10c | 265.881 |
| ZNF644 | zinc finger protein 644 | 269.136 |
| MCTP1 | multiple C2 and transmembrane domain containing 1 | 269.349 |
| ZBTB44 | zinc finger and BTB domain containing 44 | 269.637 |
| ADAM22 | ADAM metallopeptidase domain 22 | 270.135 |
| CDC27 | cell division cycle 27 | 271.049 |
| RAB11B | RAB11B, member RAS oncogene family | 272.867 |
| GOLPH3L | golgi phosphoprotein 3 like | 273.537 |
| APMAP | adipocyte plasma membrane associated protein | 274.788 |
| ELMO2 | engulfment and cell motility 2 | 275.995 |
| FNBP4 | formin binding protein 4 | 276.2 |
| VEZF1 | vascular endothelial zinc finger 1 | 276.432 |
| CTSC | cathepsin C | 276.767 |
| GXYLT1 | glucoside xylosyltransferase 1 | 277.495 |
| CASP8AP2 | caspase 8 associated protein 2 | 278.835 |
| SUGP2 | SURP and G-patch domain containing 2 | 279.763 |
| MPC2 | mitochondrial pyruvate carrier 2 | 280.14 |
| CPT1A | carnitine palmitoyltransferase 1A | 280.222 |
| MELK | maternal embryonic leucine zipper kinase | 280.36 |
| KAT6B | lysine acetyltransferase 6B | 283.69 |
| ZNF224 | zinc finger protein 224 | 283.884 |
| BCL2 | BCL2 apoptosis regulator | 284.322 |
| HIPK2 | homeodomain interacting protein kinase 2 | 284.873 |
| NDE1 | nudE neurodevelopment protein 1 | 285.613 |
| ZC3HAV1 | zinc finger CCCH-type containing, antiviral 1 | 286.289 |
| GOLGB1 | golgin B1 | 286.976 |
| TUT7 | terminal uridylyl transferase 7 | 286.992 |
| WDFY2 | WD repeat and FYVE domain containing 2 | 287.331 |
| PPIE | peptidylprolyl isomerase E | 287.45 |
| EVL | Enah/Vasp-like | 287.909 |
| TMEM9B | TMEM9 domain family member B | 289.053 |
| APC | APC regulator of WNT signaling pathway | 289.913 |
| ATF1 | activating transcription factor 1 | 290.266 |
| GINM1 | glycoprotein integral membrane 1 | 293.845 |
| TACC1 | transforming acidic coiled-coil containing protein 1 | 293.983 |
| TM2D1 | TM2 domain containing 1 | 294.54 |
| CENPJ | centromere protein J | 295.545 |
| ARRDC4 | arrestin domain containing 4 | 295.892 |
| WASF2 | WASP family member 2 | 296.093 |
| STIM1 | stromal interaction molecule 1 | 297.603 |
| CDCA2 | cell division cycle associated 2 | 297.628 |
| CENPC | centromere protein C | 298.135 |
| TMEM161B | transmembrane protein 161B | 300.188 |
| GPATCH11 | G-patch domain containing 11 | 300.76 |
| UHMK1 | U2AF homology motif kinase 1 | 300.857 |
| ZWINT | ZW10 interacting kinetochore protein | 301.522 |
| SLC35C2 | solute carrier family 35 member C2 | 302.001 |
| TRAPPC6B | trafficking protein particle complex subunit 6B | 302.554 |
| DCK | deoxycytidine kinase | 302.795 |
| CDK17 | cyclin dependent kinase 17 | 303.989 |
| LMBRD1 | LMBR1 domain containing 1 | 304.789 |
| C6orf89 | chromosome 6 open reading frame 89 | 305.261 |
| DPY19L4 | dpy-19 like 4 | 305.324 |
| KIF5B | kinesin family member 5B | 307.567 |
| ARCN1 | archain 1 | 311.279 |
| ZNF264 | zinc finger protein 264 | 311.423 |
| TGFBR1 | transforming growth factor beta receptor 1 | 312.31 |
| SFMBT2 | Scm like with four mbt domains 2 | 312.864 |
| DIAPH3 | diaphanous related formin 3 | 315.784 |
| INPP5D | inositol polyphosphate-5-phosphatase D | 315.837 |
| XYLT1 | xylosyltransferase 1 | 316.431 |
| DPY19L1 | dpy-19 like C-mannosyltransferase 1 | 316.94 |
| ATAD5 | ATPase family AAA domain containing 5 | 320.003 |
| CCNK | cyclin K | 320.799 |
| ABCA1 | ATP binding cassette subfamily A member 1 | 321.62 |
| AP3M2 | adaptor related protein complex 3 subunit mu 2 | 322.261 |
| CKS2 | CDC28 protein kinase regulatory subunit 2 | 324.859 |
| BRWD1 | bromodomain and WD repeat domain containing 1 | 324.983 |
| KATNAL1 | katanin catalytic subunit A1 like 1 | 325.194 |
| SLK | STE20 like kinase | 325.688 |
| GOLGA8A/GOLGA8B | golgin A8 family member A | 326.549 |
| BPNT2 | 3'(2'), 5'-bisphosphate nucleotidase 2 | 327.157 |
| MSL1 | MSL complex subunit 1 | 327.477 |
| HES1 | hes family bHLH transcription factor 1 | 327.73 |
| PLK4 | polo like kinase 4 | 328.303 |
| TAOK1 | TAO kinase 1 | 328.546 |
| ABITRAM | actin binding transcription modulator | 328.661 |
| C18orf25 | chromosome 18 open reading frame 25 | 329.364 |
| MGA | MAX dimerization protein MGA | 333.009 |
| NHLRC2 | NHL repeat containing 2 | 334.983 |
| POGZ | pogo transposable element derived with ZNF domain | 336.545 |
| AP1S2 | adaptor related protein complex 1 subunit sigma 2 | 338.919 |
| PAN3 | poly(A) specific ribonuclease subunit PAN3 | 338.95 |
| CASP3 | caspase 3 | 340.481 |
| TMEM65 | transmembrane protein 65 | 341.23 |
| POMK | protein O-mannose kinase | 342.99 |
| HDGF | heparin binding growth factor | 345.691 |
| RPGR | retinitis pigmentosa GTPase regulator | 345.87 |
| CHTOP | chromatin target of PRMT1 | 348.796 |
| NFAT5 | nuclear factor of activated T cells 5 | 351.097 |
| DPP8 | dipeptidyl peptidase 8 | 357.249 |
| MRE11 | MRE11 homolog, double strand break repair nuclease | 358.397 |
| FAM168B | family with sequence similarity 168 member B | 360.162 |
| RPL22 | ribosomal protein L22 | 361.433 |
| CEP135 | centrosomal protein 135 | 361.536 |
| POLK | DNA polymerase kappa | 364.144 |
| RXRB | retinoid X receptor beta | 366.163 |
| DERL1 | derlin 1 | 369.653 |
| TMX3 | thioredoxin related transmembrane protein 3 | 372.367 |
| SLC35A3 | solute carrier family 35 member A3 | 374.815 |
| TRAM1 | translocation associated membrane protein 1 | 375.568 |
| COX17 | cytochrome c oxidase copper chaperone COX17 | 377.193 |
| PSPC1 | paraspeckle component 1 | 381.482 |
| MED12 | mediator complex subunit 12 | 384.116 |
| CCDC88A | coiled-coil domain containing 88A | 385.226 |
| NCOA3 | nuclear receptor coactivator 3 | 386.381 |
| NR2C2 | nuclear receptor subfamily 2 group C member 2 | 387.736 |
| SPCS3 | signal peptidase complex subunit 3 | 390.565 |
| CENPN | centromere protein N | 391.675 |
| BLZF1 | basic leucine zipper nuclear factor 1 | 393.512 |
| SLITRK6 | SLIT and NTRK like family member 6 | 393.528 |
| TMEM131L | transmembrane 131 like | 395.074 |
| MMS22L | MMS22 like, DNA repair protein | 395.441 |
| DCAF16 | DDB1 and CUL4 associated factor 16 | 401.731 |
| SERINC1 | serine incorporator 1 | 402.463 |
| SMC1A | structural maintenance of chromosomes 1A | 403.191 |
| SMARCC2 | SWI/SNF related, matrix associated, actin dependent regulator of chromatin subfamily c member 2 | 407.121 |
| H2BC7 | H2B clustered histone 7 | 408.739 |
| PNRC1 | proline rich nuclear receptor coactivator 1 | 410.289 |
| SLC35B2 | solute carrier family 35 member B2 | 412.487 |
| DEK | DEK proto-oncogene | 418.343 |
| TASP1 | taspase 1 | 421.512 |
| FAM13B | family with sequence similarity 13 member B | 426.217 |
| KMT2D | lysine methyltransferase 2D | 427.757 |
| KIF18B | kinesin family member 18B | 430.181 |
| ATXN7 | ataxin 7 | 433.032 |
| H2BC10 | H2B clustered histone 10 | 436.271 |
| SYVN1 | synoviolin 1 | 439.872 |
| GOLT1B | golgi transport 1B | 444.412 |
| DPYD | dihydropyrimidine dehydrogenase | 445.545 |
| H2BC11 | H2B clustered histone 11 | 448.298 |
| MGAT4A | alpha-1,3-mannosyl-glycoprotein 4-beta-N-acetylglucosaminyltransferase A | 448.477 |
| TOPBP1 | DNA topoisomerase II binding protein 1 | 456.887 |
| CENPM | centromere protein M | 458.494 |
| NCAPH | non-SMC condensin I complex subunit H | 459.435 |
| SURF4 | surfeit 4 | 461.839 |
| RAD51AP1 | RAD51 associated protein 1 | 462.039 |
| ALCAM | activated leukocyte cell adhesion molecule | 462.048 |
| TMED10 | transmembrane p24 trafficking protein 10 | 464.492 |
| NSD2 | nuclear receptor binding SET domain protein 2 | 466.417 |
| ATP5MG | ATP synthase membrane subunit g | 467.481 |
| ZGRF1 | zinc finger GRF-type containing 1 | 467.96 |
| EMC7 | ER membrane protein complex subunit 7 | 475.963 |
| RAB8B | RAB8B, member RAS oncogene family | 478.45 |
| GRK3 | G protein-coupled receptor kinase 3 | 479.588 |
| SPPL2A | signal peptide peptidase like 2A | 490.735 |
| STT3B | STT3 oligosaccharyltransferase complex catalytic subunit B | 492.5 |
| MARCHF6 | membrane associated ring-CH-type finger 6 | 495.217 |
| TNPO3 | transportin 3 | 502.552 |
| TNFAIP3 | TNF alpha induced protein 3 | 509.493 |
| H1-2 | H1.2 linker histone, cluster member | 510.745 |
| PHC3 | polyhomeotic homolog 3 | 511.385 |
| RPS16 | ribosomal protein S16 | 511.687 |
| VAPB | VAMP associated protein B and C | 513.064 |
| MED12L | mediator complex subunit 12L | 519.212 |
| SOX4 | SRY-box transcription factor 4 | 520.375 |
| MGME1 | mitochondrial genome maintenance exonuclease 1 | 525.096 |
| TK1 | thymidine kinase 1 | 533.687 |
| PHIP | pleckstrin homology domain interacting protein | 533.852 |
| ARHGAP19-SLIT1 | ARHGAP19-SLIT1 readthrough (NMD candidate) | 535.165 |
| MTCH1 | mitochondrial carrier 1 | 536.874 |
| SP1 | Sp1 transcription factor | 541.208 |
| HP1BP3 | heterochromatin protein 1 binding protein 3 | 542.768 |
| PSENEN | presenilin enhancer, gamma-secretase subunit | 546.535 |
| KIFC1 | kinesin family member C1 | 549.552 |
| MBOAT1 | membrane bound O-acyltransferase domain containing 1 | 554.018 |
| UBE2A | ubiquitin conjugating enzyme E2 A | 558.495 |
| ZDHHC20 | zinc finger DHHC-type palmitoyltransferase 20 | 564.663 |
| HEY1 | hes related family bHLH transcription factor with YRPW motif 1 | 566.983 |
| TOR1AIP1 | torsin 1A interacting protein 1 | 567.895 |
| CKAP2L | cytoskeleton associated protein 2 like | 575.229 |
| UBE2M | ubiquitin conjugating enzyme E2 M | 577.28 |
| ASPM | assembly factor for spindle microtubules | 582.302 |
| H2AC13 | H2A clustered histone 13 | 604.844 |
| NSD3 | nuclear receptor binding SET domain protein 3 | 605.348 |
| FAM111B | FAM111 trypsin like peptidase B | 605.794 |
| ATAD2 | ATPase family AAA domain containing 2 | 610.741 |
| H2BC15 | H2B clustered histone 15 | 611.718 |
| TM9SF4 | transmembrane 9 superfamily member 4 | 612.302 |
| FAM72B | family with sequence similarity 72 member B | 612.582 |
| WDR33 | WD repeat domain 33 | 618.747 |
| ZFC3H1 | zinc finger C3H1-type containing | 627.624 |
| MT-ND5 | NADH dehydrogenase subunit 5 | 633.767 |
| CDK6 | cyclin dependent kinase 6 | 657.559 |
| GLG1 | golgi glycoprotein 1 | 661.171 |
| CDK1 | cyclin dependent kinase 1 | 672.197 |
| MED13L | mediator complex subunit 13L | 694.838 |
| CEP152 | centrosomal protein 152 | 696.789 |
| ELK4 | ETS transcription factor ELK4 | 701.812 |
| ABHD2 | abhydrolase domain containing 2, acylglycerol lipase | 702.152 |
| AURKA | aurora kinase A | 702.565 |
| WEE1 | WEE1 G2 checkpoint kinase | 711.304 |
| ANLN | anillin actin binding protein | 726.839 |
| ATP13A3 | ATPase 13A3 | 732.971 |
| TMEM59 | transmembrane protein 59 | 759.965 |
| H2AC14 | H2A clustered histone 14 | 775.478 |
| PRNP | prion protein | 775.931 |
| CBFB | core-binding factor subunit beta | 784.021 |
| NEMP1 | nuclear envelope integral membrane protein 1 | 784.75 |
| CKS1B | CDC28 protein kinase regulatory subunit 1B | 796.916 |
| VAT1 | vesicle amine transport 1 | 800.153 |
| CKLF | chemokine like factor | 803.718 |
| MPDU1 | mannose-P-dolichol utilization defect 1 | 815.252 |
| GUSBP2 | GUSB pseudogene 2 | 821.623 |
| CD53 | CD53 molecule | 826.823 |
| ZNF776 | zinc finger protein 776 | 839.636 |
| MED26 | mediator complex subunit 26 | 841.27 |
| SKA2 | spindle and kinetochore associated complex subunit 2 | 857.543 |
| HACD2 | 3-hydroxyacyl-CoA dehydratase 2 | 858.949 |
| EIF1B | eukaryotic translation initiation factor 1B | 864.33 |
| SACM1L | SAC1 like phosphatidylinositide phosphatase | 870.004 |
| ITM2A | integral membrane protein 2A | 893.392 |
| LAPTM5 | lysosomal protein transmembrane 5 | 909.246 |
| SPTBN1 | spectrin beta, non-erythrocytic 1 | 950.299 |
| ITM2B | integral membrane protein 2B | 966.673 |
| TYMS | thymidylate synthetase | 1014.054 |
| H2AC21 | H2A clustered histone 21 | 1051.351 |
| FAM111A | FAM111 trypsin like peptidase A | 1051.776 |
| TMEM123 | transmembrane protein 123 | 1098.508 |
| UFM1 | ubiquitin fold modifier 1 | 1131.112 |
| DAZAP2 | DAZ associated protein 2 | 1145.817 |
| B2M | beta-2-microglobulin | 1236.303 |
| TMSB10/TMSB4X | thymosin beta 4 X-linked | 1248.175 |
| CRNDE | colorectal neoplasia differentially expressed | 1286.223 |
| H1-4 | H1.4 linker histone, cluster member | 1312.587 |
| TRAT1 | T cell receptor associated transmembrane adaptor 1 | 1338.378 |
| PTPRC | protein tyrosine phosphatase receptor type C | 1344.907 |
| PLP2 | proteolipid protein 2 | 1351.705 |
| SLC38A2 | solute carrier family 38 member 2 | 1393.527 |
| MBNL1 | muscleblind like splicing regulator 1 | 1427.804 |
| SLC3A2 | solute carrier family 3 member 2 | 1431.597 |
| CHST11 | carbohydrate sulfotransferase 11 | 1610.211 |
| CXCR4 | C-X-C motif chemokine receptor 4 | 1744.499 |
| H2AC18/H2AC19 | H2A clustered histone 18 | 1815.408 |
| CD82 | CD82 molecule | 2085.209 |
| HMGB2 | high mobility group box 2 | 2221.684 |

**
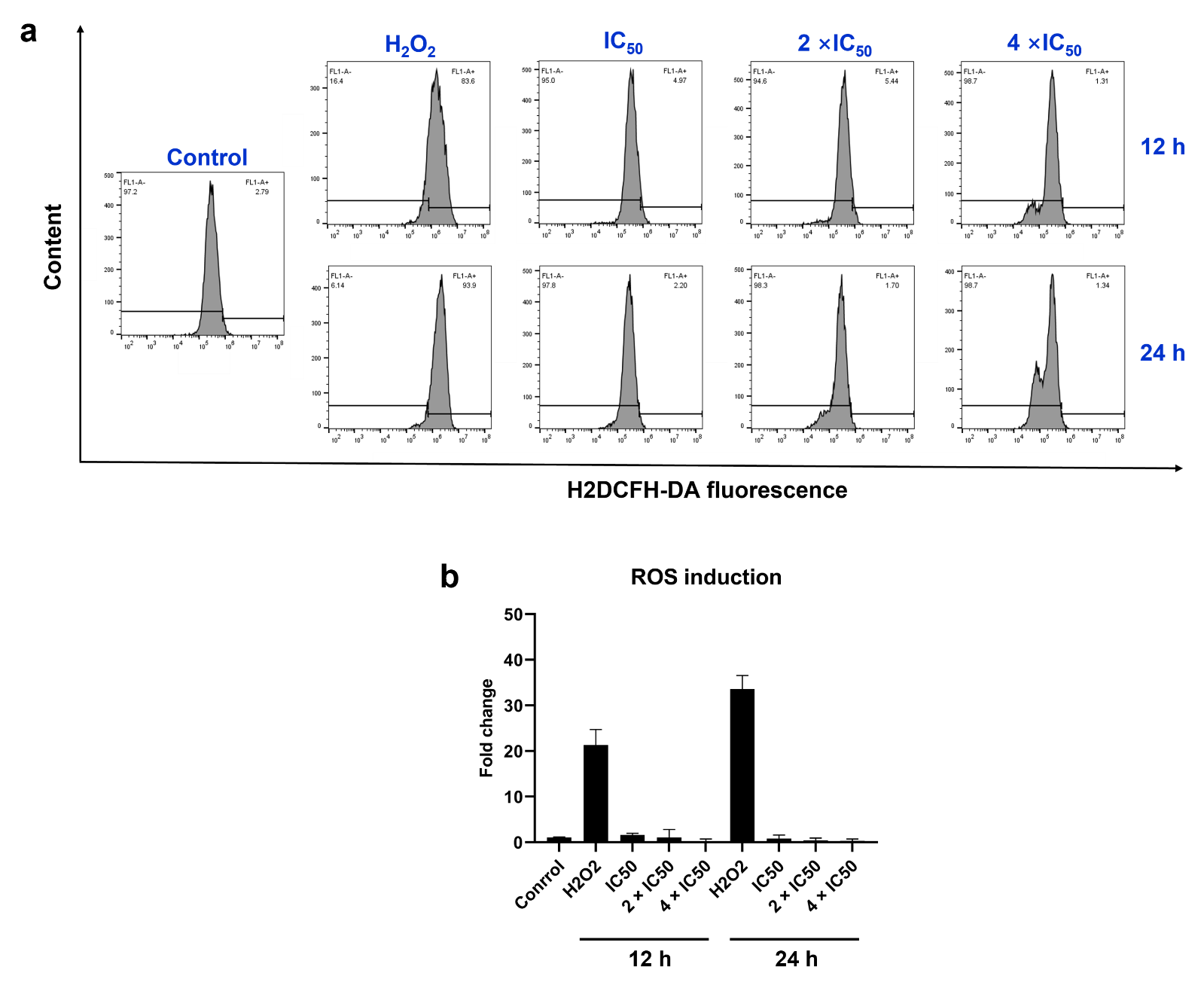
**

**Supplementary Figure S4:** Measurement of ROS upon ZINC253504760 treatment in CCRF-CEM cells. **a** Assessment of ROS with ZINC253504760 or DMSO (positive control) for 12 or 24 h, and H_2_O_2_ (positive control) for 15 min. **b** ROS quantification with fold changes of three independent experiments.

**Supplementary Table S5:** Molecular docking of ZINC253504760 and its deglycosylated form, and trametinib to MEK1 and MEK2. The lowest binding energy and the predicted inhibition constant (pKi) are shown. Each docking experiment was repeated three times

| Protein | Compounds | Lowest binding energy (kcal/mol) | pKi (µM) | Interacting amino acid (residues in H-bond are bolded) |
| --- | --- | --- | --- | --- |
| MEK1 | ZINC253504760 | -8.15 ± 0.3 | 1.22 ± 1.63 | A: ASN78, GLY79, GLY80, LYS97, HIS100, LEU101, GLU102, LEU115, ASP190, ASP208, PHE209, GLY210, SER212, LEU215, **SER218** |
|  | ZINC253504760 (deglycosylated form) | -6.26 ± 0.05 | 25.82 ± 1.95 | A: **ASN78, LYS97, ILE99, ASP190, LYS192, GLY210, MET219** |
|  | Trametinib | -10.98 ± 0.04 | 0.1 ± 0.0007 | A: LYS97, LEU115, VAL127, ILE141, ARG189, ASP208, PHE209, VAL211, **SER212**, MET219, ALA220, ARG234 |
| MEK2 | ZINC253504760 | -7.85 ± 0.54 | 2.66 ± 0.4 | A: GLU106, SER222  B: ASN82, **LYS101,** ASP212, GLY214, ARG231, SER232, TYR233 |
|  | ZINC253504760 (deglycosylated form) | -6.57 ± 0.005 | 15.37 ± 0.08 | B: **LYS101, ARG193, ASP194, ASN199, ASP121, GLY214, ILE220, MET223, ARG238** |
|  | Trametinib | -10.45 ± 0.01 | 0.2 ± 0.0003 | B: LYS101, LEU119, LEU122, VAL131, ARG193, ASP212, PHE213, GLY214, VAL215, SER216, LEU219, ILE220, ARG238 |
